# Nuclear Phosphoproteome Reveals Prolyl Isomerase PIN1 as a Modulator of Oncogene-Induced Senescence

**DOI:** 10.1016/j.mcpro.2024.100715

**Published:** 2024-01-10

**Authors:** Rodrigo Mohallem, Uma K. Aryal

**Affiliations:** 1 Department of Comparative Pathobiology, Purdue University, West Lafayette, USA; 2 Purdue Proteomics Facility, Bindley Bioscience Center, Purdue University, West Lafayette, USA

**Keywords:** fibroblast, nuclear phosphoproteome, oncogene-induced senescence, prolyl isomerase, promyelocytic leukemia nuclear body

## Abstract

Mammalian cells possess intrinsic mechanisms to prevent tumorigenesis upon deleterious mutations, including oncogene-induced senescence (OIS). The molecular mechanisms underlying OIS are, however, complex and remain to be fully characterized. In this study, we analyzed the changes in the nuclear proteome and phosphoproteome of human lung fibroblast IMR90 cells during the progression of OIS induced by oncogenic RAS^G12V^ activation. We found that most of the differentially regulated phosphosites during OIS contained prolyl isomerase PIN1 target motifs, suggesting PIN1 is a key regulator of several promyelocytic leukemia nuclear body proteins, specifically regulating several proteins upon oncogenic Ras activation. We showed that PIN1 knockdown promotes cell proliferation, while diminishing the senescence phenotype and hallmarks of senescence, including p21, p16, and p53 with concomitant accumulation of the protein PML and the dysregulation of promyelocytic leukemia nuclear body formation. Collectively, our data demonstrate that PIN1 plays an important role as a tumor suppressor in response to oncogenic ER:Ras^G12V^ activation.

## Highlights

• Phosphoproteomic landscapes of IMR90-ER:Ras^G12V^ cells during senescence are described.
• Most of the identified phosphorylated sites were preferentially PIN1 target motifs.
• Many known PML nuclear body proteins were differentially regulated.
• PIN1 depletion promotes proliferation and reduction of PML nuclear bodies in fibroblast cells.

## Graphical Abstract

**Figure:**
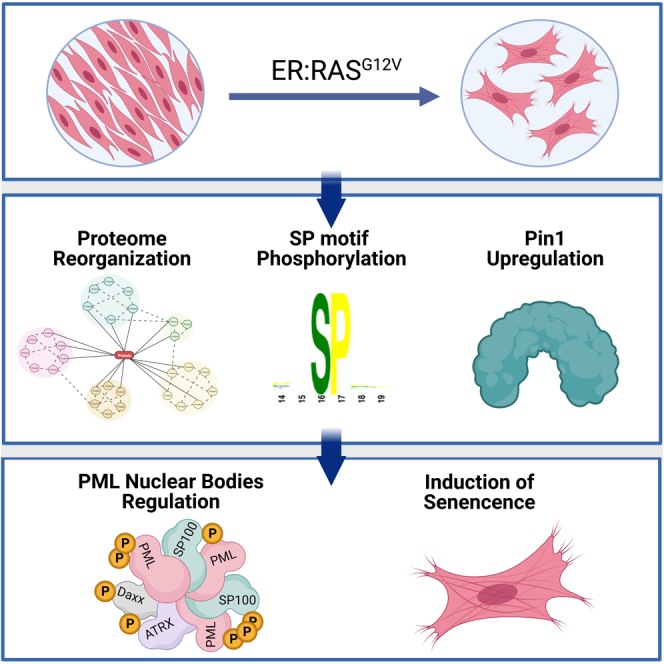

## In Brief

The molecular mechanisms underlying oncogene-induced senescence (OIS) are complex and not fully understood. In this study, we applied high-throughput nuclear proteomic and phosphoproteomic analyses of IMR90-ER:Ras^G12V^ cells after inducing senescence by the activation of ER:Ras^G12V^. This study identifies PIN1 as a modulator of OIS as the PIN1 depletion results in senescence bypass and promotes cell proliferation with concomitant reduction of PML nuclear bodies.

Cellular senescence is a state of permanent cell cycle arrest that occurs in response to genomic instability, caused by DNA damage, telomere shortening, and oncogene activation (1, 2). Senescence and cancer are highly correlated biological phenomena, as cellular senescence is the underlying mechanism of aging (3), and the degenerative changes that accompany aging constitute the single most important risk factor for cancer. Additionally, senescence acts as a strong tumor suppressor mechanism that prevents the proliferation of cells that harbor genetic aberrations (4). Unfortunately, the conditions that turn a tumor-suppressor mechanism into a tumorigenic one remain largely unknown. Therefore, understanding the biology of senescence and the mechanisms that enable cells to evade this process can provide fundamental insights into the intrinsic cellular mechanisms of organismal aging and cancer prevention. In this study, we focused on oncogene-induced senescence (OIS) triggered by the activation of oncogenic Ras to determine how the reorganization of the cellular proteome and phosphoproteome leads to the development of the senescence phenotype.

OIS entails the involvement of a multitude of proteins, including transcription factors and chromatin structural proteins (5, 6, 7). Although it is known that OIS functions through the activation of Rb and p53 pathways, it involves complex molecular networks mediated by hundreds of intermediary proteins that cooperate to establish the senescence phenotype. Despite these observations, the roles and regulations of these intermediaries and their relationship with the well-characterized tumor suppressors Rb and p53 during the progression of OIS is not well understood.

Nuclear proteins are the primary modulators of senescence (8, 9). During OIS, there is a robust shift in the nuclear architecture and subnuclear structures (10), including the promyelocytic leukemia protein–nuclear bodies (PML-NBs), the nucleolus, and Cajal bodies (CBs) (11, 12). These NBs are complex and dynamic structures that change in response to various stimuli, and recent studies have implicated their involvement in the induction of cellular senescence upon oncogenic transformation (13, 14, 15). The stability and function of many nuclear proteins including PML-NBs and CBs during OIS are mediated by posttranslational modifications (PTMs), including phosphorylation, SUMOylation, and ubiquitination (16, 17, 18, 19). However, the regulation of these PTMs in the context of Ras-induced OIS remains elusive.

In this study, we utilize recent advances in the throughput and sensitivity of proteomics technologies to analyze the complex network of acute cellular responses to Ras^G12V^-mediated senescence on a global scale. We evaluated phosphorylation dynamics of thousands of nuclear proteins including many PML-NB proteins and shed light on their roles in promoting OIS upon Ras^G12V^ activation. Further, we demonstrated that a key protein identified from our unbiased approach, peptidyl-prolyl *cis/tr*ans isomerase (PIN1), is essential for inducing cellular senescence in fibroblasts and functions by regulating the protein levels of both tumor suppressors and oncogenes. Interestingly, we identified that PIN1 is also associated with PML-NB biogenesis and function during OIS.

## Experimental Procedures

### Cell Culture

Human IMR-90 primary lung fibroblasts from the American Type Culture Collection and the IMR-90 cells infected with a vector expressing a mutant form of oestrogen receptor (ER) fused with the H-Ras^G12V^ (IMR90-ER:Ras^G12V^) were cultured in Dulbecco's modified Eagle's medium (high glucose, pyruvate) (Thermo Fisher Scientific), supplemented with 10% fetal bovine serum, and incubated at 7.5% CO_2_ and 3% O_2_. IMR90-ER:Ras^G12V^ medium was also supplemented with 200 μg/ml Geneticin (G418 sulfate) (Thermo Fisher Scientific).

### Virus Production and Viral Infections

All expression and shRNA vector plasmids were custom ordered from VectorBuilder. Packaging plasmids pCMV-VSV-G and pCMV-dR8.2-dvpr were a gift from Bob Weinberg (Addgene plasmid # 8454; http://n2t.net/addgene:8454; RRID:Addgene_8454/Addgene plasmid # 8455; http://n2t.net/addgene:8455; RRID:Addgene_8455) (20)

Lentiviral vectors were produced as previously described (21). For lentiviral transduction, cells were cultured in lentivirus containing media, supplemented with 5 μg/ml polybrene for 48 h (21). Infected cells were then selected for 5 days with 400 μg/ml geneticin (IMR90-ER:Ras) or for 2 days with 1 μg/ml puromycin (shPIN1 and scramble). For shPIN1 and scramble cells, 4-hydroxytamoxifen (4-OHT) treatment was performed immediately after the selection was completed.

### Experimental Design and Statistical Rationale

For ER:Ras^G12V^ activation, cells were treated with 100 nM (Z)-4-hydroxytamoxifen (0.01% v/v) (Millipore Sigma) in three biological replicates with 4-OHT for 0, 2, 4, and 6 days for proteomics and Western blot experiments. For the other experiments performed, cells were treated with 100 nM (Z)-4-hydroxytamoxifen (0.01% v/v) (Millipore Sigma) for 6 days and vehicle control cells were treated with equivalent volume of MeOH. Statistical analysis was performed using a one-way (global and phospho proteomics) or two-way (shPIN1 proteomics) ANOVA. Multiple comparisons were corrected by Šidák test.

### Nuclear Extraction

Cells were washed three times with 5 ml 1 × PBS (4 °C), collected by scraping and centrifuged. For nuclear enrichment (22), cell pellets were resuspended in 500 μl of extraction buffer (supplemented with protease and phosphatase inhibitors), flash-frozen at −80 °C for 10 min, and allowed to thaw on ice for 40 min. Samples were then centrifuged at 1150*g* for 10 min at 4 °C. Supernatant (cytosolic fraction) was discarded; pellets were washed with extraction buffer and again centrifuged at 1150*g* for 15 min. Supernatant was removed, the pellets (nuclear fraction) were resuspended in 300 μl lysis buffer (supplemented with protease and phosphatase inhibitors), and sonicated (6 cycles of 3 s pulses, 5 s interval). Protein concentration was measured with the bicinchoninic acid assay (Thermo Fisher Scientific).

### Sample Preparation for Mass Spectrometry Analysis

Equivalent volumes of 500 μg of total protein were precipitated overnight at −20 °C with four volumes of acetone. Samples were then centrifuged at 17300*g* for 15 min, supernatant was removed, and protein pellets were quickly dried in a vacuum centrifuge.

Samples were resolubilized in 50 μl of 8 M urea and incubated at room temperature for 30 min. Disulfide bonds were reduced with 10 mM DTT for 45 min at 37 °C. Samples were then cooled to room temperature. Cysteines were alkylated with 20 mM iodoacetamide for 45 min in the dark, at room temperature. After alkylation, DTT was added to the samples (5 mM) and incubated at 30 °C for 20 min. Samples were then diluted with 230 μl 50 mM ammonium bicarbonate containing 10 μg trypsin (Thermo Fisher Scientific), and 15 μl acetonitrile (ACN), and incubated at 37 °C for 2 h. An additional 10 μg trypsin were then added in 5 μl ammonium bicarbonate, and samples were further incubated for 2 h at 37 °C. The digestion was halted by the addition of 0.3 μl formic acid (FA). Peptides were then desalted with the pierce peptide desalting spin columns (Thermo Fisher Scientific). A total of 20 μg of peptides from each sample were separated at this step for the global analysis. Phosphopeptide enrichment was then carried out using the remainder of the samples with the PolyMac spin tips (Tymora analytical), following manufacturer’s recommendations.

### Mass Spectrometry Analysis

Dried samples were reconstituted in 3% ACN, 0.1% FA and separated using a 26 cm nanoflow Aurora Series UHPLC packed emitter columns, by reversed-phase chromatography in a Dionex UltiMate 3000 RSLC system (Thermo Fisher Scientific). Global analysis was performed with a 280-min gradient method, with a flowrate of 150 nl/min. Briefly, samples were injected with 2% mobile phase solution B (80% ACN with 0.1% FA in water). Mobile phase B was then increased to 5% at 5 min, and linearly increased, reaching 30% B at 230 min. B was then increased up to 60% until 265 min, at which point it was reverting to 2% B until the end of the run. Phosphopeptides and shPIN1/scramble samples were separated in a 160-min gradient. Samples were injected in 2% B and increased in a linear fashion until 27% B was reached at 110 min, 40% B at 125 min, and 100% B at 135 min. The concentration of B was then held at 100% for 10 min before returning to 2% B and maintained at 2% B until the end of the run. Mass spectrometry (MS) analyses were performed in the Orbitrap Fusion Lumos Tribrid Mass Spectrometer, with the orbitrap detector, with a MS1 resolution of 120,000 and MS2 resolution at 7500. Quadrupole isolation was set to “true.” The scan range used was between 375 and 1500 *m/z*. The radio frequency lenses was set to 30%, AGC target was set to “standard,” with a maximum injection time of 50 s and 1 microscan. A dynamic exclusion duration of 60 s was used, with exclusion of isotopes. The instrument was operated on data-dependent acquisition mode with the “cycle time” of 3 s between scans. Higher energy collisional dissociation was used for fragmentation, with higher energy collisional dissociation collision energy set to 30%. We also used an tandem mass spectrometry (MS/MS) maximum injection time of 50 s and 1 microscan.

### Development and Analytical Validation Targeted MS Assays/Measurements

For multiple reaction monitoring (MRM) method development, the Skyline software (v22.2) (skyline.ms) MacCoss Lab Software) was used. All peptides used for the assay were selected using previously reported transitions available in the SRM Atlas (Complete Human SRMAtals database). At least four proteotypic tryptic peptides (no missed cleavages) per protein were then imported into Skyline, and the transition list with collision energy parameters were generated. Both doubly and triply (2+ and 3+) charge peptides were used. The scheduled transition lists were then created for the final assay. The MRM analyses were performed with a TSQ Endura Triple Quadrupole Mass Spectrometer (Thermo Fisher Scientific) with the Flex ESI-interface in a SRM mode in positive polarity. The MS analysis was conducted with the spray voltage set at 2400 V, and the transfer capillary temperature was set at 275 °C. The MRM transitions were acquired with a cycle time of 0.8 s, Q1 resolution of 0.7 (full width at half maximum) and Q3 resolution of 1.2. The collision energy for each transition differed and was obtained with the Skyline software. Cysteine carbamidomethylation was set as a fixed modification, and *y*-ions were used as the product ions. Peaks containing at least three transitions, with consistent retention time across all samples were considered for further analysis. Peak areas for each peptide were used for a one-way ANOVA, followed by Šídák’s multiple comparisons tests across treatment groups. The detailed transition list and collision energy values used can be found in supplemental Table S12. The liquid chromatography (LC) setup used was the same as described in the previous section, run in a 60-min gradient. Briefly, samples were injected in 2% B and increased in a linear fashion until 27% B was reached at 40 min, 45% B at 45 min, and 100% B at 50 min. The concentration of B was then held at 100% for min before returning to 2% B and maintained at 2% B until the end of the run.

### Protein Identification and Quantification

Raw MS/MS data were searched against the UniProt (23) *Homo sapiens* database (UniProt Proteome, downloaded on April 21st, 2021; 99,013 entries, including isoforms) using the MaxQuant platform (Ver 2.0.3.1; www.maxquant.org). Trypsin enzymes were selected for specific digestion, with up to two missed cleavages (or four for phosphoproteomics). Variable modifications were set for “methionine oxidation” and for phosphoproteomics “STY phosphorylation;” and “carbamidomethyl” was set as a fixed modification. A false discovery rate of 1% was used for both peptides and proteins identification. Additionally, 10 ppm was selected as the main search peptide tolerance value, and 20 ppm was set for the MS/MS match tolerance. Peptide quantitation was performed using “unique plus razor peptides. The MaxQuant output files were processed and analyzed using the Perseus biostatistics platform (maxquant.net/perseus) for statistical analysis. “contaminants,” “reverse,” and “only identified by site” proteins were filtered out, and label-free quantitation (LFQ) intensity values were Log2 transformed. All downstream analyses were performed after further data filtering to retain only proteins identified by an at least two MS/MS counts and detected in minimum valid values in at least one treatment group. Missing values were imputed based on the normal distribution of LFQ values. Intensity values and localization probability scores were used for phosphoprotein analysis. The raw phospho STY data file was filtered for proteins with a localization probability ≥0.75, and two valid intensity values in at least one-time point. Missing values were again imputed with values drawn from the normal distribution. Statistical significance was inferred based on ANOVA tests. Proteins with a *p* value ≤0.01 for global and *p* value ≤0.05 for phospho and knockdown datasets were considered significantly regulated. Gene ontology was performed using ShinyGO (bioinformatics.sdstate.edu/go/) (24) and Metascape (metascape.org) (25) online softwares, with only biological processes (BPs) (gene ontology) selected for annotation, membership, and enrichment. Moonlight (26), Phosphopath (27) and PhosR (28) packages were used in R, following developer’s recommendations. Libraries were generated by compiling protein–protein interaction annotations with PML (for PML-NB) from the BioGRID (29), CORUM (30), database of interacting proteins: the database of interacting proteins (31), IntAct (32), MINT (33), the nuclear protein database (34), and UniProt databases. MRMs were processed in Skyline.

### Confocal Microscopy

Cell collection, cytocentrifugation, cell fixation, and immunofluorescence were performed as previously described (21, 35). The PML protein was probed with the anti-PML protein antibody [C7] (ab96051; Abcam), and the goat anti-mouse secondary antibody, alexa fluor plus 488 (A32723TR; Thermo Fisher Scientific). Nuclei were stained with 4′,6-diamidino-2-phenylindole (Millipore Sigma). Samples were imaged with the Nikon A1Rsi confocal microscope, and PML-foci were quantified using ImageJ (imagej.net/ij) (36).

### Western Blots

Western blots were performed as previously published (37). Proteins were probed with the following antibodies: PML polyclonal antibody (# PA5-29614; Thermo Fisher Scientific), p53 mAb (DO-7) (# MA5-12557; Thermo Fisher Scientific), p21 mAb (R.229.6) (# MA5-14949; Thermo Fisher Scientific), recombinant anti-Pin1 antibody (ab192036, Abcam), monoclonal anti-β-actin antibody (# A5441, Sigma-Aldrich).

### SA-β-GAL Assays

SA-β-GAL assay was performed with the senescence β-galactosidase staining kit (#9860; Cell Signaling Technology), following manufacturer's recommendations.

## Results

### Nuclear Proteome and Phosphoproteome Landscapes of OIS upon ER:Ras Activation by 4-OHT

Changes in the nuclear proteome and phosphoproteome of senescent cells remain largely unexplored, despite evidence that the nucleus and subnuclear structures are extensively reorganized during OIS, and have been suggested to contribute in developing the senescent phenotype (9, 38, 39). To determine what proteins may drive the nuclear rearrangement observed in OIS, we generated a model of OIS utilizing human diploid fibroblast IMR90 cells, transduced with a lentiviral vector for the stable expression of the 4-OHT–inducible, fusion protein ER:Ras^G12V^. ER:Ras^G12V^ protein expression was confirmed by LC-MS/MS analysis (data not shown). To induce OIS, unsynchronized ER: Ras^G12V^ mutant cells were treated with 100 nM 4-OHT, and collected at two (D2), four (D4), and 6 days (D6) after initial treatment (D0). The induction of senescence phenotype was confirmed by senescence-associated β-galactosidase (SA-β-gal) staining assay, after 6 days of 4-OHT or methanol (MeOH; vehicle control) treatment (supplemental Fig. S1*A*). With confirmation of the suitability of our model, and that the selected treatment dose and duration is sufficient to induce OIS, we proceeded with a temporal nuclear proteome analysis.

**Fig. 1:**
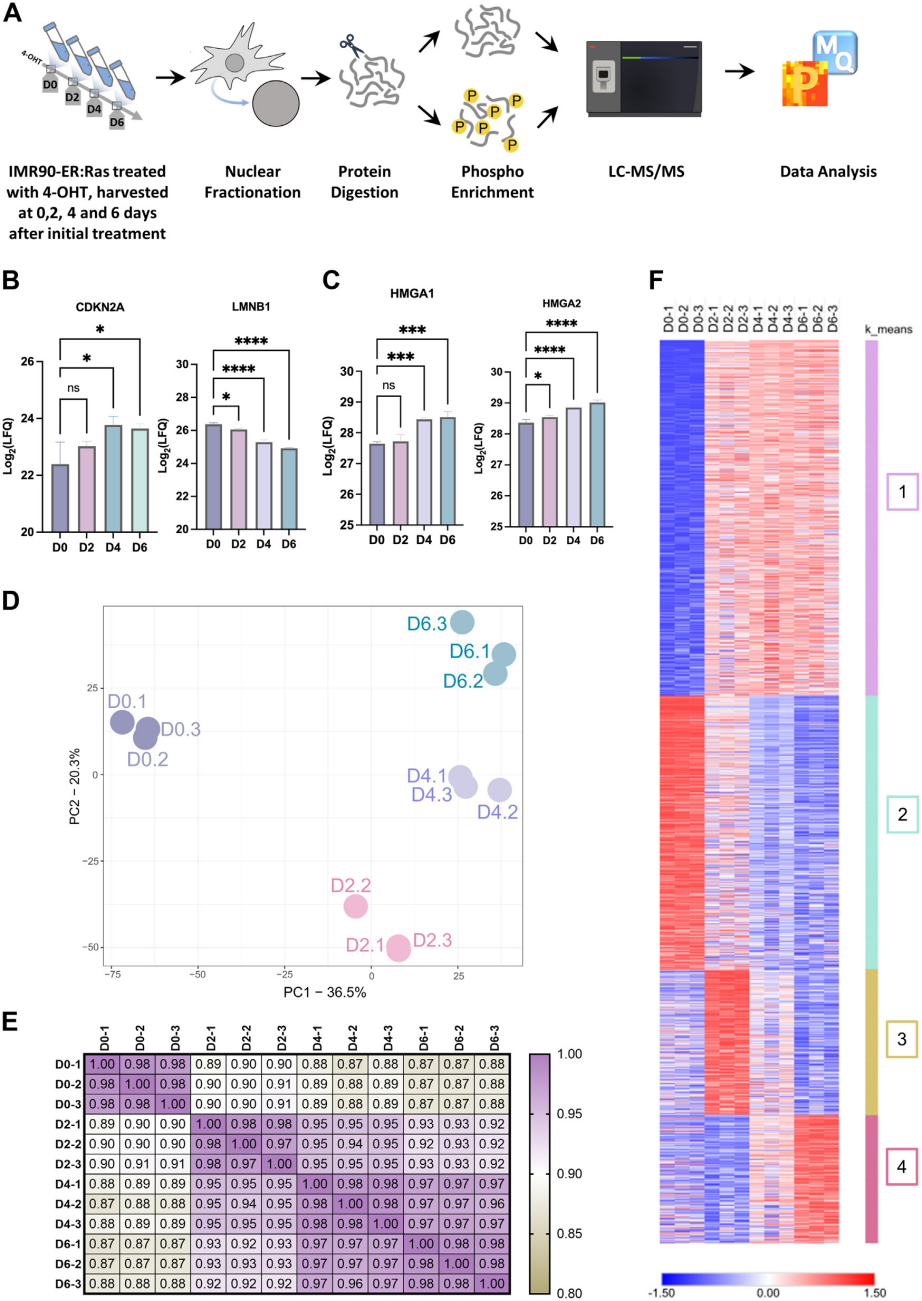
**Temporal analysis of the IMR-90 ER:Ras proteome during OIS.***A*, *graphical representation* of the experimental workflow. Cells treated in three biological replicates for 0, 2, 4, and 6 days had their nucleus fractionated, and nuclear proteins were extracted. Proteins were digested, and phosphopeptides were enriched prior to LC-MS/MS. Raw LC–MS/MS data were then processed with the MaxQuant (www.maxquant.org) and Perseus (maxquant.net/perseus) software. *B*, the senescence hallmark proteins CDKN2A (p16) and LMNB1, and the (*C*) senescence-associated heterochromatin foci (SAHF) proteins HMGA1 and HMGA2 are significantly regulated during the progression of OIS (n.s. = not significant, ∗*p* < 0.05, ∗∗*p* < 0.01, ∗∗∗*p* < 0.01, ∗∗∗∗*p* < 0.001; one-way ANOVA followed by Šidák correction). *D*, PCA plot of all replicates analyzed in global proteomics. Time points are indicated by *color*, and individual samples are annotated. *E*, Pearson’s correlation plot of significantly regulated proteins. *Color scale* indicates correlation score. *F*, heat map depicting the *z*-scored Log2(LFQ) values of all significant proteins at each time point. Proteins were clustered by K-means clustering. Each cluster is indicated by a unique number and given assorted colors. *Red hue* indicates upregulated proteins, and *blue hue* indicates downregulated proteins. ER, oestrogen receptor; LC, liquid chromatography; LFQ, label-free quantitation; OIS, oncogene-induced senescence; PCA, principal component analysis.

Crude nuclear fraction of IMR90-ER:Ras^G12V^ cells were (supplemental Fig. S1*B*) prepared for LC-MS/MS analyses, in biological triplicates, as previously described (40). All MS data were acquired in an Orbitrap Fusion Lumos MS (Thermo Fisher Scientific) in data dependent acquisition mode. Raw LC-MS/MS files were processed in the MaxQuant (37, 41) and Perseus (42) platforms (Fig. 1*A*).

In our global analysis, we identified a total of 81,058 peptides, mapped to 6264 protein groups. Filtering for proteins quantified in at least two biological replicates (LFQ > 0), we obtained 4960 protein groups from which 2013 were significantly different based on a one-way ANOVA (supplemental Tables S1 and S2 and supplemental Fig. S1, *C* and *D*). In our phosphoproteomic analysis, we identified 19,248 phosphosites from 23,648 phosphopeptides with distinct multiplicities of which 10,673 were class I phosphosites (phosphosites with a localization probability > 0.75) also quantified in at least two replicates. Of those, 5641 phosphosites were significantly regulated during OIS (supplemental Tables S3 and S4).

### ER:Ras^G12V^ Activation Promotes Changes in the Nuclear Proteome in a Time-Dependent Manner

It has been suggested that activation of p16^INK4a^, an inhibitor of CDK4 and CDK6, maintains durable growth arrest (43). Indeed, our results show the levels of the tumor suppressor p16 (CDKN2A) significantly increased after D4 of 4-OHT exposure, compared to D0. Lamin B1, loss of which is known to serve as biomarker of senescence (44), was significantly downregulated at D2 compared to D0, with an observed continuous decrease in its protein levels at D4 and D6, consistent with previous reports (44, 45) (Fig. 1*B*). We also detected a significant upregulation in the senescence-associated heterochromatin foci proteins HMGA1 and HMGA2 (46), further confirming that oncogenic Ras activation by 4-OHT treatment promotes OIS through activation or inhibition of well-characterized senescent-associated biomarkers.

Principal-component analysis of all significant proteins revealed that all biological replicates within each treatment group clustered together (Fig. 1*D*). As evidenced by the distance between the control D0 and 4-OHT treated D2, D4, and D6 samples captured by PC1, Ras^G12V^ activation induced significant changes in the nuclear proteome of IMR90 cells, which is exacerbated by treatment duration, depicted by the increased distance between the later timepoints relative to D0 (Fig. 1*D*). Additionally, Pearson Correlation analysis also indicated a clear segregation between 4-OHT–treated samples, with D4 and D6 time points highlighting remarkably high correlation scores of 97% or higher (Fig. 1*E*).

As indicated by the heatmap (Fig. 1*F*), the changes in significantly regulated proteins could be explained broadly by four distinct clusters. Cluster 1 represents proteins upregulated at D2, with subsequent plateauing throughout the remainder of the time points. This cluster is composed of proteins involved in mRNA processing, over-representing BP such as “translation,” “regulation of mRNA metabolic process,” and “regulation of telomerase RNA localization to CB.” In contrast, cluster 2 contains proteins whose levels steadily decreased following the time progression of 4-OHT treatment, which are involved in “cellular response to growth factor stimulus” and “transmembrane receptor protein tyrosine kinase signaling pathway,” suggesting a downregulation of proteins downstream of the Ras signaling pathway (supplemental Fig. S2).

Cluster 3 is characterized by an acute increase in protein levels at D2, which gradually decreases to levels below basal at D6. This cluster is enriched for BP also involved in mRNA processing, DNA replication and cell cycle progression, including “cell division,” “DNA-template transcription, elongation,” “regulation of cell cycle,” and “DNA repair”. These observations suggest that Ras^G12V^ activation elicits a primary response that induces cell cycle progression, a response that is subsequently diminished, which may explain the development of cellular senescence at D6 (7). Cluster 4 displays a direct opposite trend compared to cluster 3, and contains proteins involved primarily in metabolic processes (supplemental Fig. S2).

### ER:Ras^G12V^ Activation by 4-OHT Differentially Regulates Putative Nuclear Body Proteins

**Fig. 2:**
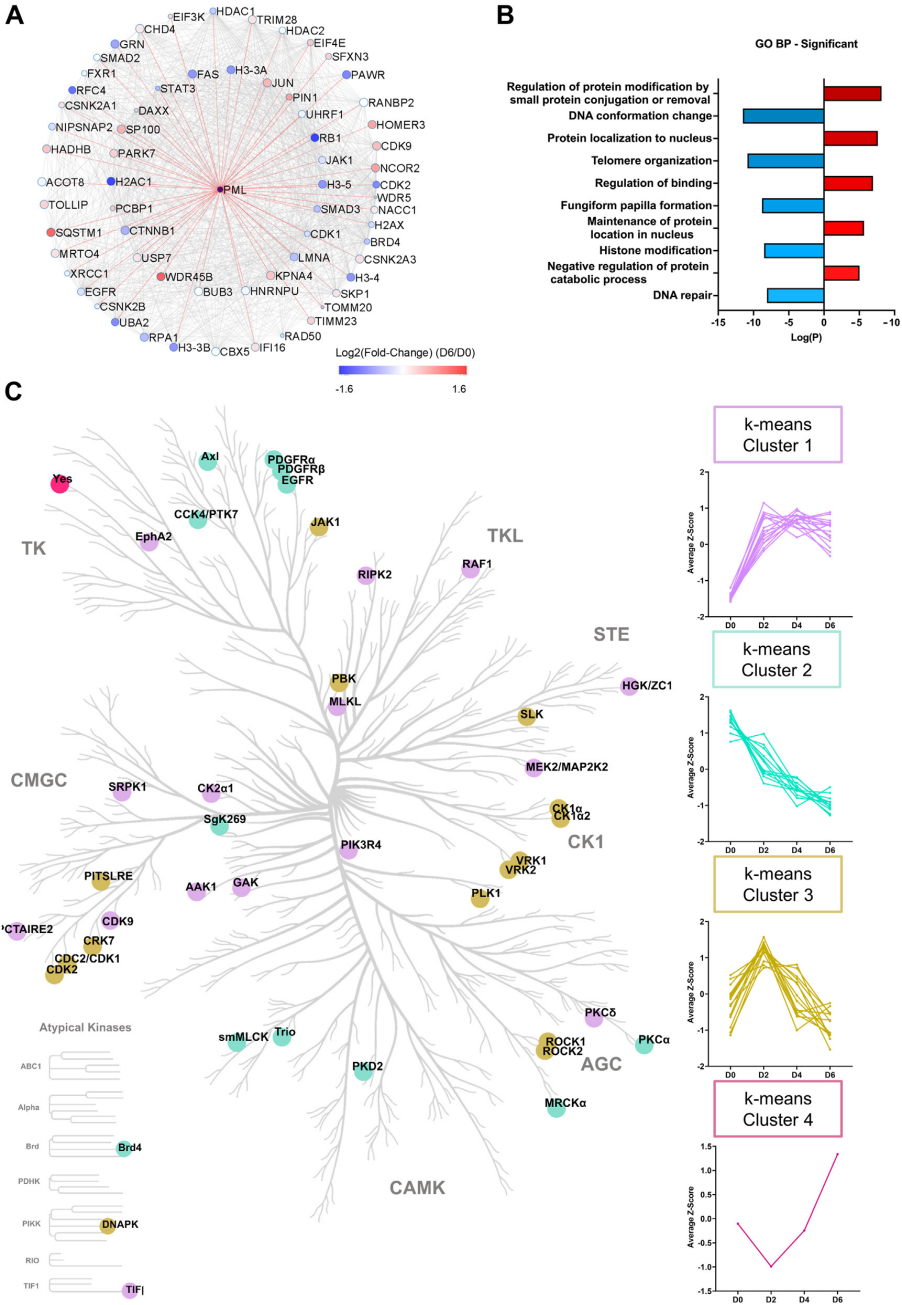
**Dynamic regulation of PML-NB proteins and kinases during OIS.***A*, significantly regulated PML-NB proteins depicted *via* putative protein–protein interactions (see supplemental Table S7). *Red hue* indicates upregulated proteins, and *blue hue* indicates downregulated proteins at D6 of 4-OHT treatment. *B*, GO enrichment of significantly regulated biological processes in which regulated PML-NB proteins are involved in. *C*, kinase tree representation of significantly regulated kinases. *Colors* represent clusters based on protein regulation patterns observed in the heatmap depicted in Fig. 1*F*. 4-OHT, 4-hydroxytamoxifen; GO, gene ontology; OIS, oncogene-induced senescence; PML-NB, promyelocytic leukemia protein–nuclear body.

Nuclear bodies, including PML-NBs, are highly dynamic structures, which serve as hubs for protein–protein and protein–RNA interactions (47, 48, 49). Recent evidence suggests an interplay between cellular senescence and PML-NBs (38, 50), therefore, an unbiased omics-based approach to study the regulation of PML-NB proteins during OIS is critical for identifying more direct relationships between these proteins and the process of OIS. To determine the effect of Ras^G12V^-induced OIS on PML-NB proteins, we compiled data on known PML-NB proteins and their putative protein–protein interactions across several available databases (see Experimental Procedures for details) and constructed a PML-NB protein library. Utilizing this library, we interrogated our proteomics dataset and identified 202 putative PML-NB–interacting proteins from which 59 proteins were significantly regulated in response to 4-OHT treatment (Fig. 2*A* and supplemental Table S7). To our knowledge, this is the largest collection of putative PML-NB protein dataset in a single experiment. However, we stress that we identified hundreds of PML-NB proteins based on curated databases and their identification does not refer to their direct molecular interactions in this study. For simplicity, these putative PML-NB proteins will be referred to as PML-NB proteins.

Interrogating our nuclear proteome dataset with the curated PML-NB dataset library identified several known nuclear body interactor proteins that were significantly regulated and are recognized as important players involved in cellular senescence. Downregulated proteins of interest included proteins involved in the control of cell cycle progression, such as the cyclin-dependent kinases CDK1 and CDK2 and protein RB1. Our data also reveals the downregulation of casein kinase II subunit beta (CK2b; CSNK2B), a kinase whose upregulation is known to evade senescence and promote cancer progression (51). Nonetheless, CK2b has also been demonstrated to promote senescence escape *via* the phosphorylation and subsequent ubiquitination and degradation of PML (51). CK2 also activates JAK/STAT3 pathway (52), and interestingly, both STAT3 and JAK1 were downregulated at D6. On the other hand, the constitutive PML-NB proteins SP100 and DAXX, as well as p16, were upregulated after Ras^G12V^ activation. The prolyl isomerase PIN1 protein was also significantly upregulated (Fig. 2*A*). Upregulated PML-NB proteins were involved in BPs such as “regulation of protein modification by small protein conjugation or removal,” whereas downregulated proteins were enriched for processes that are tightly linked with the development of the senescence phenotype, such as “DNA conformational change,” “telomere organization,” and “histone modifications” (Fig. 2*B*).

### ER:Ras^G12V^ Activation by 4-OHT Dysregulates the Nuclear Phosphoproteome

When disrupted, the Ras signaling pathway leads to uncontrolled phosphorylation events that ripples through tightly interconnected signaling networks, leading to the dysregulation of cellular functions, including triggering OIS in IMR90 cells (7, 53). A kinase tree shows significantly regulated kinases in our nuclear dataset that are grouped according to their regulatory profile, shown in Figure 1*F* (Fig. 2*C*). Kinases that are directly up/downstream of abnormal Ras signaling, such as EGFR, PDFR, and PKC showed a steady decline in their abundance levels. Kinases from the same family appear to have similar regulatory patterns during OIS. The Yes kinase was the single kinase to fall under cluster 4 (Fig. 2*C*).

**Fig. 3:**
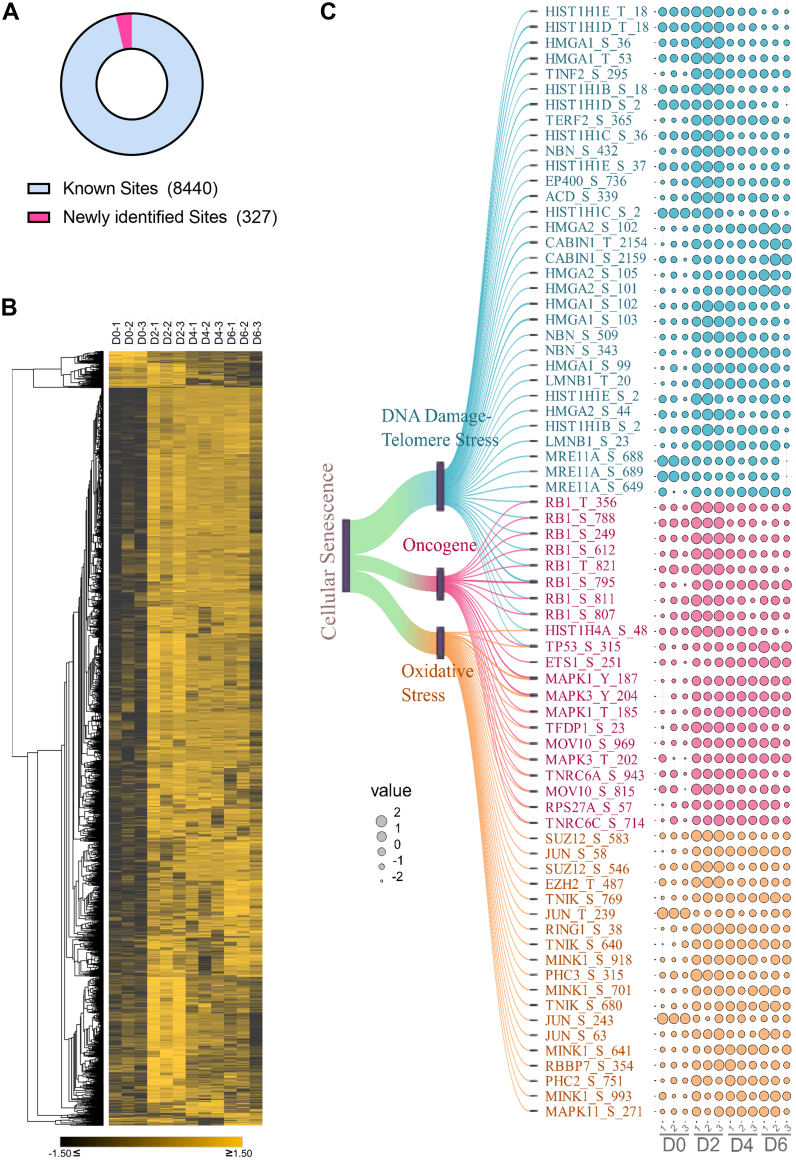
**Temporal regulation of the IMR-90 ER:Ras phosphoproteome during OIS.***A*, *pie chart* highlighting the distribution between the number of newly identified class I sites and known class I sites in our data, according to the PhosphoSitePlus database. *B*, *heatmap representation* of class I phosphosites significantly regulated in our analysis. Hue represents the *z*-score normalized Log2(label-free quantitation) values. *Yellow hue* indicates sites upregulated and *black* represents sites downregulated upon 4-OHT treatment. Sites were clustered based on one minus Pierce correlation. *C*, *heatmap representation* of enriched proteins in the Reactome pathways “DNA damage, telomere stress–induced senescence,” “oncogene-induced senescence,” and “oxidative stress–induced senescence,” all listed under the “cellular senescence” pathway. 4-OHT, 4-hydroxytamoxifen; ER, oestrogen receptor; OIS, oncogene-induced senescence.

To investigate the consequent changes in protein phosphorylation, we conducted a nuclear phosphoproteome analysis, which identified 10,673 class I phosphosites with distinct multiplicities, 327 of which are not included in the current PhosphoSitePlus database (54). Thus, we may have revealed the phosphorylation at these sites for the first time (Fig. 3*A* and supplemental Table S4). These new sites included proteins involved in the STAT3 and EGF/EGFR signaling pathways, indicating a potential biological relevance of these sites in driving OIS (supplemental Fig. S3, *A* and *B*).

We also observed an overall increase in the phosphorylation level of a majority of the significantly phosphorylated proteins (Fig. 3*B*). Upon phosphorylation at D2, most phosphosites intensity levels remained unchanged, contrasting with sites that showed reduced phosphorylation at D2 compared to D0 in which their phospho levels steadily decreased over the course of the treatment. We also observed a significant upregulation in the phosphorylation levels of several proteins involved in cellular senescence (Fig. 3*C*).

### Prediction of Kinase Activity During ER:Ras^G12V^ Activation by 4-OHT

**Fig. 4:**
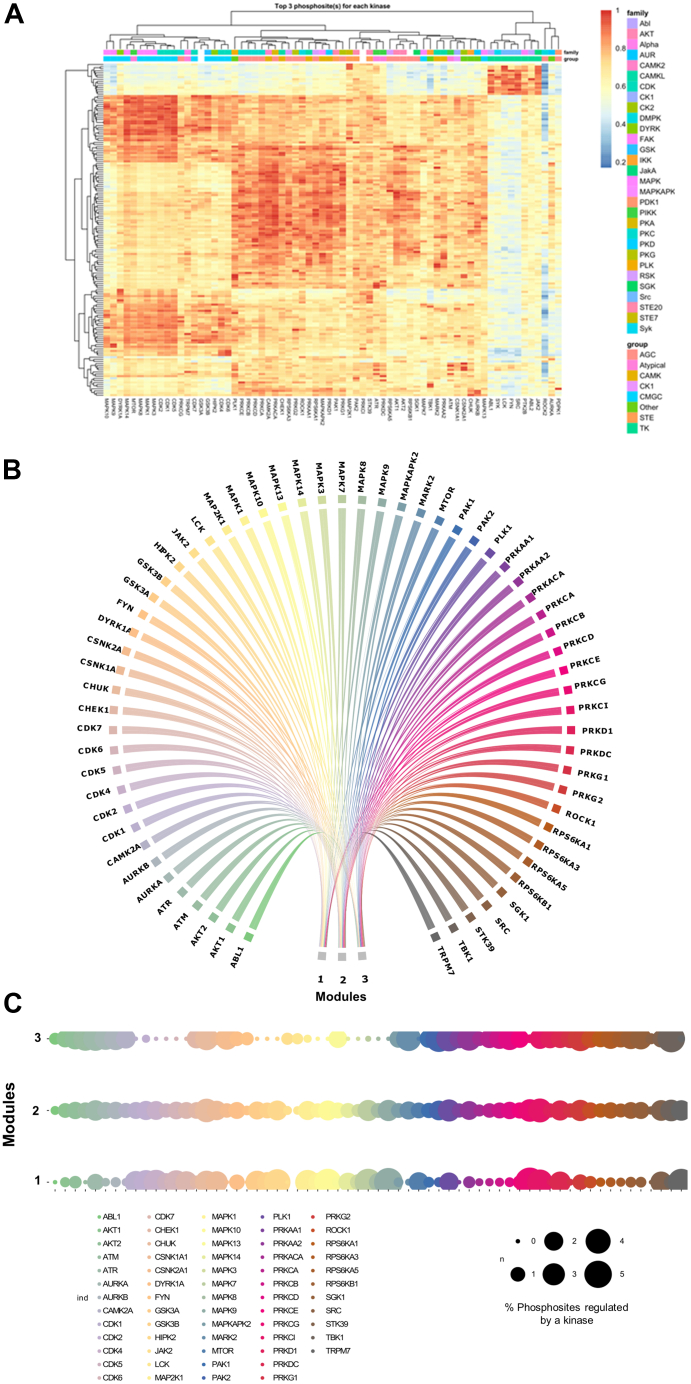
**Regulatory profile of the IMR-90 ER:Ras kinome during OIS.***A*, *heatmap representation* of enriched kinases based on upregulated phosphosites compared with D0, and their top three regulated sites. Hue represents enrichment scores. *B*, *circle plot* highlighting kinases enriched for each module, numbered 1 to 3. *C*, Signalome map depicting the percentage of phosphosites regulated by each enriched kinase in each of the modules depicted in Figure 4*B*. ER, oestrogen receptor; OIS, oncogene-induced senescence.

Changes in Ras-mediated signaling could be caused by the dysregulation of protein kinases. Therefore, we explored the potential kinases that had their activity impacted during OIS. As changes in kinase activity cannot be solely explained by changes in their protein levels, we utilized significantly regulated phosphosite dataset to expand on the regulated kinome and predict specific kinases that had an increased activity, using the PhosR tool (28). We plotted the combined kinase-substrate scores for the top three phosphosites for each enriched kinase as a heatmap (Fig. 4*A*). We obtained 63 kinases from 30 families enriched from our analysis. Their predicted kinase-substrate score is explained by three different modules, indicating their differential regulatory properties and target phosphosites (Fig. 4, *B* and *C*). Interestingly, we observed that the serine/threonine-protein kinase PRKC kinases, including PRKCD which has been previously implicated in transforming growth factor-beta–induced senescence (55), regulate a high percentage of phosphosites across all three clusters. Based on these findings, we suggest that the PRKs, in combination with other kinases significantly enriched in our analysis, including mTOR, MAPK, ATM, and ATR kinases, are likely involved in inducing OIS in fibroblast cells upon oncogenic Ras activation.

### Putative PML-NB Proteins are Hyperphosphorylated During ER:Ras^G12V^ Activation by 4-OHT

**Fig. 5:**
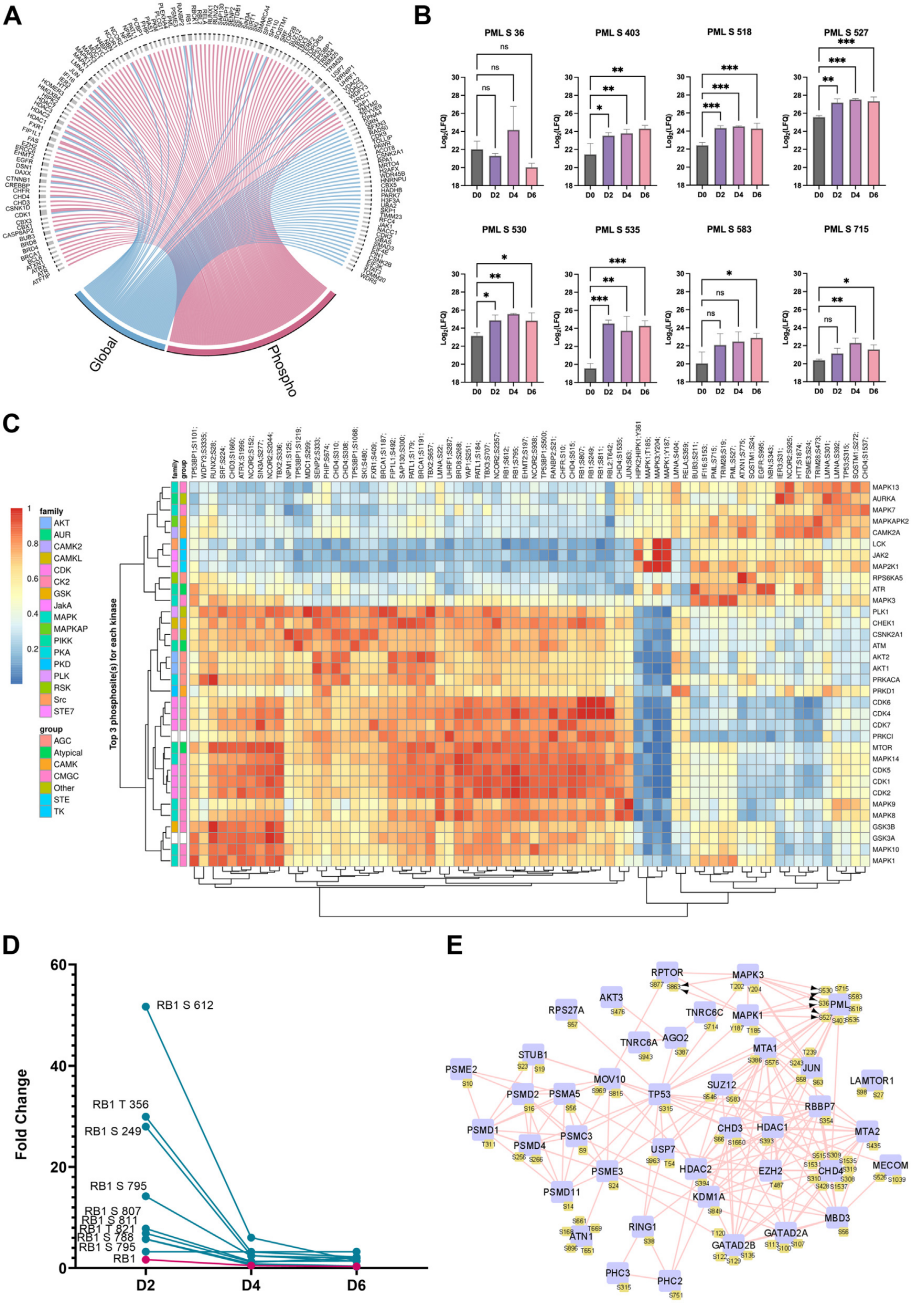
**PML-NB phosphorylation dynamics during OIS.***A*, *circle plot* depicting putative PML-NB proteins identified in our global and phospho datasets (see supplemental Table S9). *B*, *bar graph* representation of the eight PML phosphosites significantly regulated during OIS. Hues represent each time point. (n.s. = not significant, ∗*p* < 0.05, ∗∗*p* < 0.01, ∗∗∗*p* < 0.01, ∗∗∗∗*p* < 0.001; one-way ANOVA followed by Šidák correction). *C*, *heatmap representation* of enriched kinases based on phospho PML-NB proteins and their top three regulated sites. *D*, protein Rb1 phosphorylation during the progression of OIS. Phosphorylation levels at each time point are compared to D0. *Blue hue* represents phosphosites, and *pink hue* represents overall protein levels. *E*, significantly regulated phosphosites involved in the MAPK signaling pathway. OIS, oncogene-induced senescence; PML-NB, promyelocytic leukemia protein–nuclear body.

To investigate the roles of PML-NB proteins during OIS, we analyzed their phosphorylation levels during OIS. We found that phosphorylation levels of several PML-NB proteins are uniquely regulated after 6 days of 4-OHT treatment. Although some PML-NB–interacting proteins are regulated at both global and phospho levels, several were only changed at phospho levels (Fig. 5*A* and supplemental Table S9). Specifically, the protein PML was regulated by phosphorylation, with increased phosphorylation levels at seven out of eight different phosphorylated sites identified (Fig. 5*B*).

To identify kinases that preferentially target PML-NB proteins upon Ras activation, we again performed a PhosR kinase enrichment on upregulated PML-NB protein phosphosites. Our results suggest upregulated activity of 34 different kinases that potentially target PML-NB proteins during OIS. PML sites S527 and S715 were among the top three sites for phosphorylation by MAPK3. These sites were also predicted to be phosphorylated by MAPK1, ATR, and AURKA. AURKA was also enriched for p53 S315. Phosphorylation of this site has previously been shown to induce E2F1-mediated nuclear retention and transcriptional activation of p53, as well as increase in p21 expression (56).

Additionally, the protein RB1 was a target for phosphorylation at multiple sites by several of the enriched kinases. RB1 was highly phosphorylated at D2, with phosphorylation sites reaching close to 60-fold higher than D0 (Fig. 5*C*). The phosphorylation levels of these sites then decreased at D4 and D6. These observations are in line with the known regulatory effects of retinoblastoma protein in which its hyperphosphorylation regulates E2F activity and cell cycle progression (57). The initial inactivation of retinoblastoma protein by phosphorylation at D2 may enhance proliferative processes, while later dephosphorylation at D4 and D6 may reactivate RB1 to inhibit cell cycle progression (Fig. 5*D* and supplemental Fig. S4).

To gain insight into the phosphorylated pathways of PML-NB proteins and their potential interactors, we utilized the PhosphoPath tool (27) and generated biological networks utilizing our phosphoproteomics dataset. Consistent with the high kinase-substrate scores obtained for several MAP kinases, “MAPK signaling pathway” was a significantly enriched network, where PML phosphorylation on S36, S527, and S530 by MAPK1 and MAPK3 was evidenced (Fig. 5*E*). Other enriched pathways include “regulation of TP53 activity through acetylation” and “PTEN regulation” (supplemental Fig. S5, *A* and *B*).

### Involvement of PIN1 in Senescence Induced by ER:Ras^G12V^ Activation by 4-OHT Ras

After the kinase enrichment analysis, we investigated if there were any phosphorylation motifs overrepresented among the regulated phosphosites. We discovered two distinct sequence motifs, containing an RxxS or an serine-proline, that were represented over 500 times in our data, identified using an arbitrary cut-off value determined after repeated analysis with the MoMo software (momo-sysbio.gforge.inria.fr) (58) (supplemental Fig. S5*C*).

Serine-proline is the substrate motif for the ERK1/2 kinases, which are activated downstream of the Ras signaling pathway. Notably, the pSP/pTP motif is also the target sequence for the protein PIN1. PIN1, a peptidyl-prolyl *cis–trans* isomerase, catalyzes the isomerization of the peptide bond that links a phospho serine or phospho threonine to an adjacent proline. This structural change has a myriad of consequences in its target’s function, localization, protein–protein interactions, and culminates in several changes in cellular processes (59, 60).

**Fig. 6:**
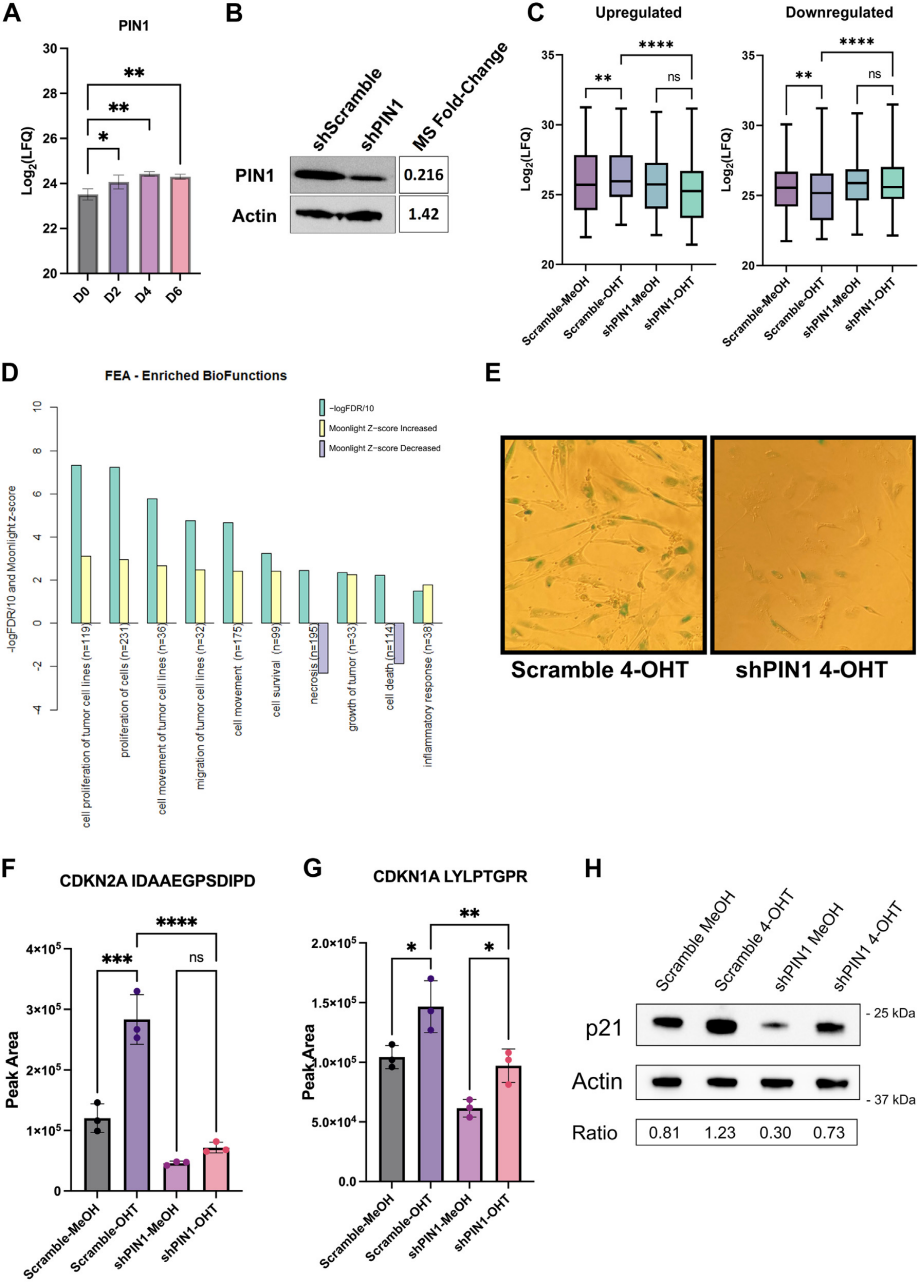
**PIN1 acts as a tumor suppressor protein.***A*, Pin1 fold-change in response to 4-OHT treatment at each time point studied. (n.s. = not significant, ∗*p* < 0.05, ∗∗*p* < 0.01, ∗∗∗*p* < 0.01, ∗∗∗∗*p* < 0.001; one-way ANOVA followed by Šidák correction). *B*, Western blot analysis of Pin1 shRNA–mediated knockdown, compared with the fold change calculated based of MS analysis. *C*, significantly regulated PIN1 target sites (one-way ANOVA followed by Šidák correction). *D*, significantly regulated biofunctions predicted by the Moonlight R package (see supplemental Table S11). *E*, senescence phenotype observed with SA-β-gal staining after 6 days of 4-OHT treatment. *F*, MRM analysis of p16 (CDN2A) protein levels and (*G*) p21 (CDN1A) proteins levels in scramble and shPIN1 IMR90-ER:RAS cells treated with MeOH or 4-OHT (one-way ANOVA followed by Šidák correction). *H*, Western blot analysis of p21 protein levels after 4-OHT or MeOH treatment of Scramble or shPIN1 cells. Quantification results show normalized band intensity relative to actin, relative to scramble-MeOH. 4-OHT, 4-hydroxytamoxifen; ER, oestrogen receptor; MRM, multiple reaction monitoring; MS, mass spectrometry.

Given the significant upregulation of PIN1 in senescent cells, with an approximate two-fold increase in its protein levels after D4 of 4-OHT treatment (Fig. 6*A*), as well as the fact that several PML-NB proteins were significantly phosphorylated at PIN1 target sites, including p53 S36, S315, and S518 (supplemental Fig. S6*A*), we hypothesized that PIN1 contributes to OIS induction. To test this hypothesis, we performed a shRNA-mediated PIN1 knockdown (shPIN1) in IMR90-ER:Ras^G12V^. We verified the efficacy of PIN1 knockdown *via* Western blot and LC-MS/MS analysis, both demonstrating a decrease in PIN1 levels by about 4-fold compared to the scramble control (Fig. 6*B*). With the efficacy of the knockdown confirmed, we proceeded with a global nuclear proteomics experiment, with cells collected after 6 days of 4-OHT or MeOH treatment. We identified a total of 5362 proteins, from which 697 were significantly regulated based on a two-way ANOVA analysis.

Using our phosphoproteomics results, we filtered for all proteins that contained a pSP/pTP motif. This protein list was then used to identify potential PIN1 target proteins in our shPN1 nuclear proteomics, to study how ablation of PIN1 affected its targets regulatory patterns in response to Ras^G12V^ activation. Our observations could be categorized into two groups. The first proteins that were downregulated after 4-OHT treatment compared to MeOH control in the scramble background. These suffered no significant changes after PIN1 ablation. Similarly, upregulated proteins in the 4-OHT treatment group compared to MeOH control in the scramble background also showed no significant changes after PIN1 knockdown (Fig. 6*C* and supplemental Table S10).

To visualize the effects of PIN1 knockdown, we plotted all significantly regulated proteins based on two-factor (Ras activation and shPIN1) ANOVA into a heatmap using k-means clustering and defined seven distinct clusters that could explain the differential regulatory patterns present in this dataset (supplemental Figs. S7 and S8). To determine the major biological processes of each cluster’s proteins, we performed WikiPathway enrichment analysis.

Cluster 1 contained proteins that were primarily downregulated in shPIN1-4OHT and upregulated in scramble-4-OHT and presented “senescence and autophagy in cancer” as the second highest significantly enriched pathway. This pathway was also enriched in cluster 5, which had a similar regulatory pattern. In contrast, clusters 2 and 3 indicate the upregulation of proteins in shPIN1-4-OHT compared to scramble-4-OHT–treated cells. Pathways enriched in this cluster include those involved in tumorigenesis and metabolism. Volcano plot of significantly regulated proteins, particularly between shPIN1 and Scramble cells, both treated with 4-OHT, showed that shPIN1 induced both significant downregulation and upregulation of several proteins, with a symmetric distribution, implying complex regulatory effects promoted by PIN1 (supplemental Fig. S6*B*).

Based on the pathway enrichment analysis, we hypothesized that PIN1 may act as a tumor suppressor protein in IMR90-ER:Ras^G12V^ cells and that its knockdown enables evasion of OIS. To determine whether the biological functions affected by PIN1 knockdown are also linked to known BPs in cancer, we performed a functional enrichment analysis with the MoonlighR package (26), which enriches known BPs in different cancer types based on differentially regulated genes. Enrichment analysis of significantly regulated proteins identified “cell proliferation of tumor cell lines” and “proliferation of cells” with highest enrichment scores. Processes involved in dysregulation of cell mobility and inflammation were among the upregulated functions, whereas “necrosis” and “cell death” were downregulated (Fig. 6*D* and supplemental Table S11). Corroborating the Moonlight analysis, we identified that the minichromosome maintenance complex protein MCM2, which are necessary for cell cycle progression and is a marker for proliferating cells (61) was upregulated following PIN1 depletion (supplemental Fig. S6*C*). Taken together, these results demonstrate that shPIN1 likely induces cell proliferation and bypasses cellular senescence.

**Fig. 7:**
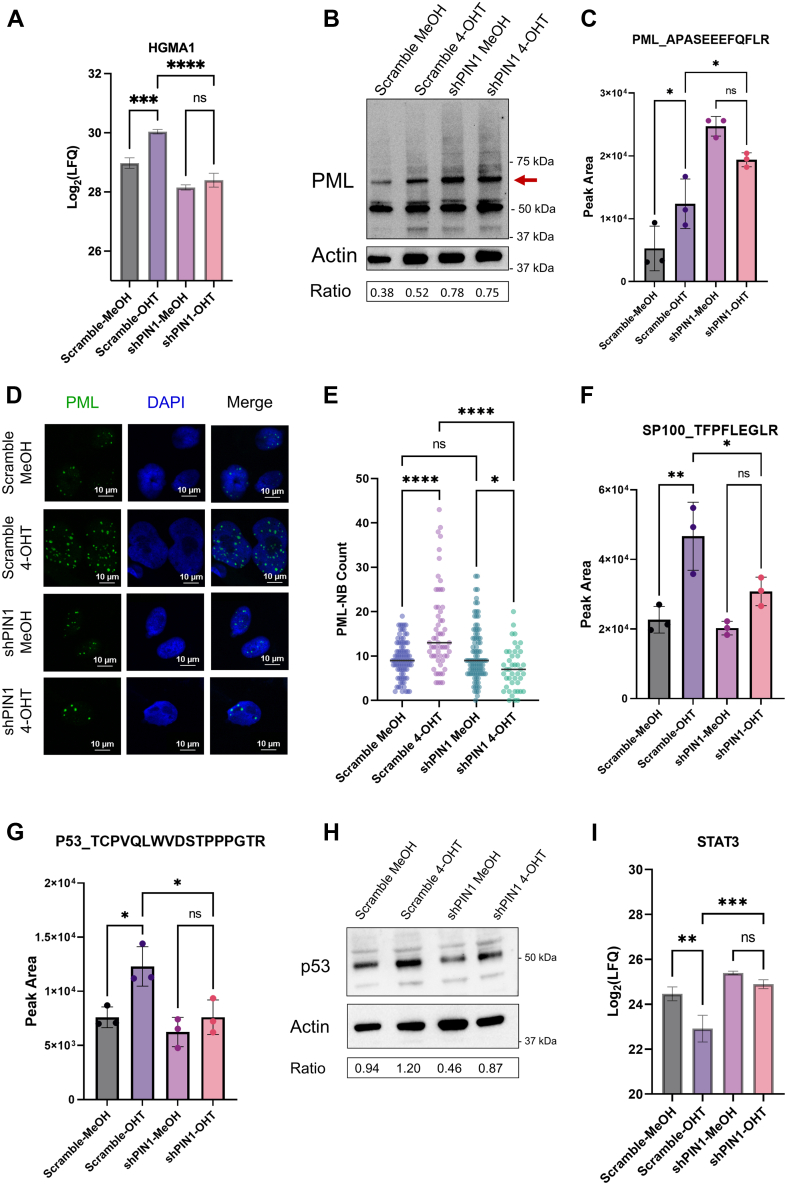
**PML-NB are regulated by PIN1 during OIS.***A*, HGMA1 is downregulated upon PIN1 knockdown. n.s. = not significant, ∗*p* < 0.05, ∗∗*p* < 0.01, ∗∗∗*p* < 0.01, and ∗∗∗∗*p* < 0.001; one-way ANOVA, followed by Šidák correction. *B*, Western blot analysis of PML protein levels after 4-OHT or MeOH treatment of scramble or shPIN1 cells. Quantification results show normalized band intensity (marked with *red arrow*) relative to actin, normalized to scramble-MeOH. *C*, MRM analysis of levels of PML in scramble and shPIN1 cells treated with MeOH or 4-OHT. (one-way ANOVA followed by Šidák correction). *D* and *E*, PML nuclear body numbers are significantly decreased upon 4-OHT treatment in shPIN1 cells compared to the scramble control. (one-way ANOVA followed by Tukey’s multiple comparison tests). *F*, MRM analysis of levels of SP100 and (*G*) p53 in scramble and shPIN1 cells treated with MeOH or 4-OHT. (one-way ANOVA followed by Šidák correction). *H*, Western blot analysis of p53 protein levels in response to shPIN1 after 6 days of 4-OHT treatment (*I*) STAT3 protein levels upon PIN1 knockdown. (one-way ANOVA followed by Šidák correction). 4-OHT, 4-hydroxytamoxifen; MRM, multiple reaction monitoring; OIS, oncogene-induced senescence; PML-NB, promyelocytic leukemia protein–nuclear body.

Indeed, SA-β-gal assay showed that PIN1-depleted cells have decreased β-gal staining compared to scramble control cells (Fig. 6*E*). Furthermore, several senescence markers and cell cycle regulator proteins were dysregulated compared to scramble, including the proteins p16 and p21. MRM analysis showed a remarkable decrease in p16 protein levels after PIN1 depletion, with no significant increase in p16 in response to 4-OHT treatment (Fig. 6*F*). p21, on the other hand, showed decreased levels in shPIN1 background, although upregulation of the protein was still observed in response to Ras^G12V^ activation (Fig. 6, *G* and *H*). Interestingly, we also observed that the senescence-associated heterochromatin foci protein HGMA1 was also downregulated in response to PIN1 depletion, and its levels were no longer affected by 4-OHT treatment (Fig. 7*A*). In fact, simultaneous depletion of both HGMA1 and p16 has been shown to increase senescence bypass (46), corroborating our initial hypothesis.

### Prolyl-Isomerase PIN1 is Necessary in PML-NB Biogenesis During OIS

Based on the increased phosphorylation of PML-NB proteins, including several sites that are potential PIN1 targets, we further examined the roles of PIN1 in regulating the PML-NB structures during OIS. MRM analysis indicated upregulation of the protein PML by more than 2-fold in response to PIN1 knockdown, consistent with Western blot results (Fig. 7, *B* and *C*). Based on these results, we hypothesized that the PIN1 depletion dysregulates PML-NB formation, given the known recruitment of PIN1 to PML-NB and that PML is a substrate of PIN1. To test this hypothesis, we utilized immunochemistry to quantify the number of PML-NB foci, and to determine the effects of PIN1 and Ras^G12V^ activation in PML-NB biogenesis and disassembly.

In scramble cells treated with 4-OHT, we observed a significant increase in PML-NB when compared to MeOH (Fig. 7, *D* and *E*). The number of PML-NB foci was similar between scramble and PIN1 knockdown cells both treated with MeOH; however, 4-OHT–treated shPIN1 cells had a significant decrease in PML-NB foci, both in comparison with shPIN1-MeOH and scramble-4-OHT (Fig. 7, *D* and *E*).

The observed decrease in PML-NB foci in 4OHT-shPIN1 cells correlates with a lack of regulation of SP100 levels, which was increased in response to 4-OHT in scramble background cells (Fig. 7*F*).

p53, another key protein in driving OIS phenotype, had an increased signal in 4-OHT–treated scramble cells compared to MeOH treated scramble cells; however, following PIN1 depletion, p53 protein levels remained unaffected (Fig. 7, *G* and *H*). Therefore, we propose that PIN1 may function as a key mediator of PML-NB during OIS and is necessary for the upregulation of tumor suppressor genes in response to Ras^G12V^ activation.

## Discussion

Here, we presented the most comprehensive analysis of the nuclear (phospho)proteome during OIS, a highly relevant cellular mechanism of tumor suppression. Identification of distinct regulatory profiles of nuclear proteins and phosphoproteins in response to ER:RAS activation *via* 4-OHT treatment led to novel findings about the roles and regulation of nuclear proteins and substructures, such as PML-NBs. We report significant changes in PML-NB and proteins, at both protein and PTM levels, including a significant increase in tumor suppressor proteins, such as p53, p16, and significant decrease in cell cycle proteins, such as CDK1, CDK2, together with the dephosphorylation of Rb1 at specific sites, corroborating with the induction of cellular senescence.

We also showed that the principal component of PML-NBs, the PML protein, was hyperphosphorylated in response to Ras^G12V^ activation (Fig. 5*B*). The phosphorylation of PML at different sites reported in this study have been correlated with distinct regulatory effects. For example, phosphorylation at S518 has been shown to induce PIN1-mediated isomerization and subsequent PML degradation (62) (Fig. 5*B*). Interestingly, most of the phosphorylated sites identified, including S36, S403, S518, S527, and S535, have been shown to promote PML sumoylation (63). Phosphorylation of S403-S535 have also been correlated with AURKA activity during the cell cycle (63, 64). Our results suggest that AURKA is a putative kinase that targets not only PML at S527 and S715 but is also strongly enriched for p53 S315 and LMNA S301 and S392 (Fig. 5*C*).

PTMs, including phosphorylation and acetylation, play a key role in the regulation of p53 function (65). Interestingly, p53 S315 is a target site for PIN1. PIN1 has been proposed to induce a conformational change in the p53 protein, which leads to its transactivation and stabilization (66, 67). Indeed, when we depleted IMR90-ER: Ras^G12V^ of PIN1, we observed a decrease in p53 protein levels, which is further exacerbated during OIS, suggesting that p53 function is reliant on the activity of PIN1 (Fig. 7, *G* and *H*). Our findings support a previous study that highlighted a downregulation of p53 levels in Ras^G12V^ cells after PIN1 knockdown (68). In this report, the authors suggested that PIN1 isomerization induces stabilization of p53 by monoubiquitination and activation of proapoptotic pathways. Indeed, after shPIN1, we observed a downregulation of cell death and necrosis pathways upon Ras^G12V^ activation (Fig. 6*D*). Furthermore, p53 isomerization by PIN1 induces p53 acetylation at K373 and K382, which is crucial for p21 transcription (69). Here, we demonstrated that PIN1 knockdown leads to a significant decrease in p21 levels, even after OIS is triggered (Fig. 6, *G* and *H*). Thus, our results reveal a regulatory role of PIN1 in which p53 isomerization at S315 is likely crucial for p53 stabilization and transactivation during cellular senescence and point to a tumor-suppressive role of PIN1 during OIS. Based on our results and on previous reports showing that p53 acetylation at K382 dictates its recruitment to PML-NBs in response to oncogenic Ras (70), we hypothesize that during senescence, p53 phosphorylation induces its isomerization by PIN1, which then triggers p53 acetylation and consequent colocalization with PML-NBs, which further stabilizes the p53 protein. However, p53 acetylation and localization to the PML-NB during OIS under our experimental conditions need to be further investigated.

Another protein that may contribute to the observed diminished p53 levels is STAT3. STAT3 protein levels were significantly higher in response to PIN1 depletion during OIS than scramble (Fig. 7*I*). Previous reports have demonstrated that in fibroblasts, STAT3 binds to the p53 promoter, repressing p53 transcription (71). In corroboration with these observations, L1CAM, a protein that has been correlated with increased tumor initiation through the activation of the STAT3 signaling cascade (72) was primarily detected in shPIN1 cells, with a 30-fold increase in its levels during OIS (supplemental Fig. S6*D*)

Despite previous reports showing that the knockdown of PIN1 leads to lower levels of cell proliferation and induction of senescence markers (73, 74, 75), with a vast majority of such studies being conducted in cancer cell models, our results show a protective role of PIN1 in the context of OIS. Our observation are in line with reports providing evidence that PIN1 is involved in cell cycle progression (76, 77), cell proliferation (78), and the knockout of PIN1 has been linked with increased genomic instability in fibroblasts (79). PIN1 ablation was also shown to enhance oncogene-induced transformation in murine fibroblasts (80). Furthermore, the regulatory roles PIN1 occupies upon Ras^G12V^ activation may differ in the context of OIS, due to the activation of the MAP kinases, and subsequent extensive phosphorylation of pSP and pTP motifs, which also serve as PIN1 target motifs. It is interesting to note that the tumor-suppressive effects of PIN1 have been correlated with a WT p53 status (81).

Together, these results shed light on a potential mechanism by which PIN1 depletion leads to cellular proliferation, senescence bypass, and potential tumor initiation. Concomitantly, we also report the dysregulation of PML-NB formation during OIS after PIN1 knockdown (Fig. 7, *B*–*F*). PIN1 knockdown resulted in a significant decrease in PML-NB foci. In contrast, PIN1 knockdown increased accumulation of the protein PML, likely due to dysregulation of phosphorylation dependent PML isomerization by PIN1. We provide strong evidence that PIN1 is necessary for the upregulation of SP100 and proper PML-NB assembly. PML-NB are fundamental subnuclear structures for the development of cellular senescence, thus, the reduction of PML-NB foci in shPIN1 cells during OIS may explain the observed attenuation of the senescence phenotype. Taken together, our study showed the potential involvement of PTM derived PIN1-p53 regulatory axis in the modulation of OIS in fibroblast cells and highlighted many mediators that play an integral role in developing OIS phenotype.

## Data Availability

All Raw LC–MS/MS data are deposited in the MassIVE data repository (massive.ucsd.edu/). MASSIVE-ID: MSV000090326.

Annotated spectra for the global dataset can be accessed on MS Viwer using the key cdone0uzvz or by using the following URL: https://msviewer.ucsf.edu/prospector/cgi-bin/mssearch.cgi?report_title=MS-Viewer&search_key=cdone0uzvz&search_name=msviewer.

Annotated spectra for the phospho dataset can be accessed on MS Viewer using the key qkuxpbzwwh or by using the following URL: https://msviewer.ucsf.edu/prospector/cgi-bin/mssearch.cgi?report_title=MS-Viewer&search_key=qkuxpbzwwh&search_name=msviewer.

Further information and requests for resources and reagents should be directed to and will be fulfilled by the lead contact, Uma K Aryal (uaryal@purdue.edu).

This study did not generate new unique reagents.

## Supplemental Data

This article contains supplemental data.

## Conflict of interest

The authors declare no competing interests.
