# Supplementary material for: Nuclear Phosphoproteome Reveals Prolyl Isomerase PIN1 as a Modulator of Oncogene-Induced Senescence
Source: Mol Cell Proteomics. 2024 Jan 10;23(2):100715. doi: 10.1016/j.mcpro.2024.100715 (PMC10864342; doi:10.1016/j.mcpro.2024.100715)
Supplement: Supplemental Figures S1–S8 [file mmc1.docx]

**Nuclear phosphoproteome reveals** **Prolyl Isomerase PIN1 as a modulator of oncogene-induced senescence**

Rodrigo Mohallem^1,2^ & Uma K. Aryal^1,2^

^1.^ Department of Comparative Pathobiology, Purdue University, West Lafayette, USA

^2.^ Purdue Proteomics Facility, Bindley Bioscience Center, Purdue University, West Lafayette, USA

| Figure S1 | Time-resolved nuclear proteomics of IMR90-ER:Ras as they undergo OIS |
| --- | --- |
| Figure S2 | Protein regulatory patterns and corresponding GO biological processes |
| Figure S3 | GO-BP of Newly identified phosphosites |
| Figure S4 | Dynamic regulation of Rb1 during cellular senescence |
| Figure S5 | Nuclear phosphoproteomics of IMR90-ER:Ras |
| Figure S6 | Pin1 regulated proteins |
| Figure S7 | shPIN1 proteome |
| Figure S8 | shPIN1 proteome, continued |

**Supplementary figures**

**Supplementary Tables**

| Table S1 | MaxQuant output – Global analysis (ProteinGroups) |
| --- | --- |
| Table S2 | Perseus statistical analysis -Global dataset |
| Table S3 | MaxQuant output – Phospho analysis |
| Table S4 | Perseus statistical analysis – Phospho dataset |
| Table S5 | shPIN1 dataset – MaxQuant output (ProteinGroups) |
| Table S6 | shPIN1 dataset – Perseus statistical analysis |
| Table S7 | List of putative PML nuclear body proteins |
| Table S8 | List of putative Cajal body proteins |
| Table S9 | List of putative PML nuclear body phosphoproteins |
| Table S10 | List of PIN1 target proteins |
| Table S11 | Moonlight proteins - functional enrichment analysis |
| Table S12 | MRM peptides transition lists |

**
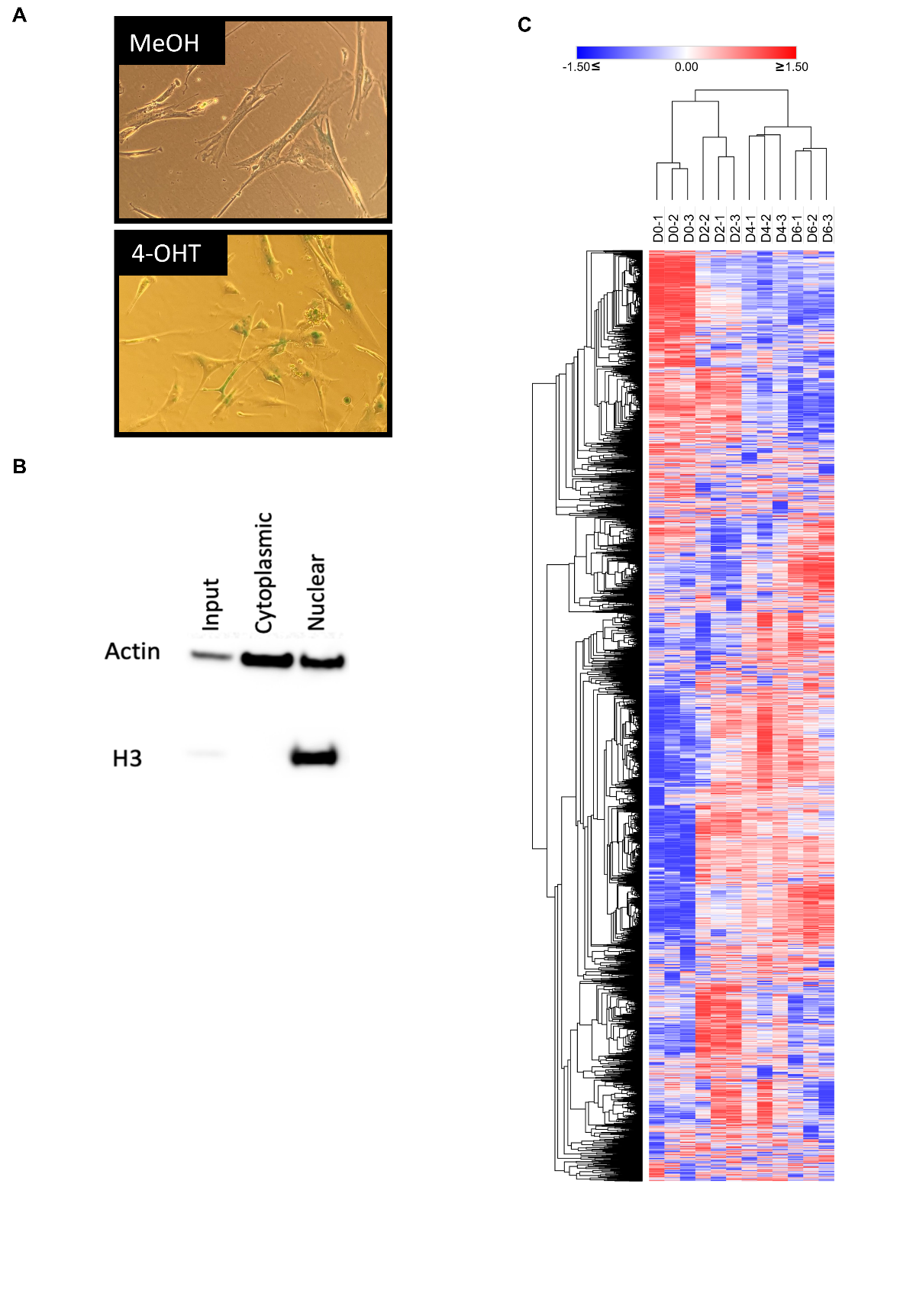
**

**Figure S1.** Time-resolved nuclear proteomics of IMR90-ER:Ras as they undergo OIS. (**A**) IMR90-ER:RasG12V cells were treated with 4-OHT or MeOH for 6 days, and the senescence phenotype was observed with SA-β-gal staining. (**B**) Western blotting of nuclear enrichment. Histone H3 was used as a marker for nuclear purification, and actin was used as a loading control. (**C**) . (**D**) Heat map depicting the Z-scored Log2(LFQ) values of all proteins identified at each timepoint. Proteins were clustered based on one minus Pierce correlation. Red hue indicates upregulated proteins, and blue hue indicates downregulated proteins.

**
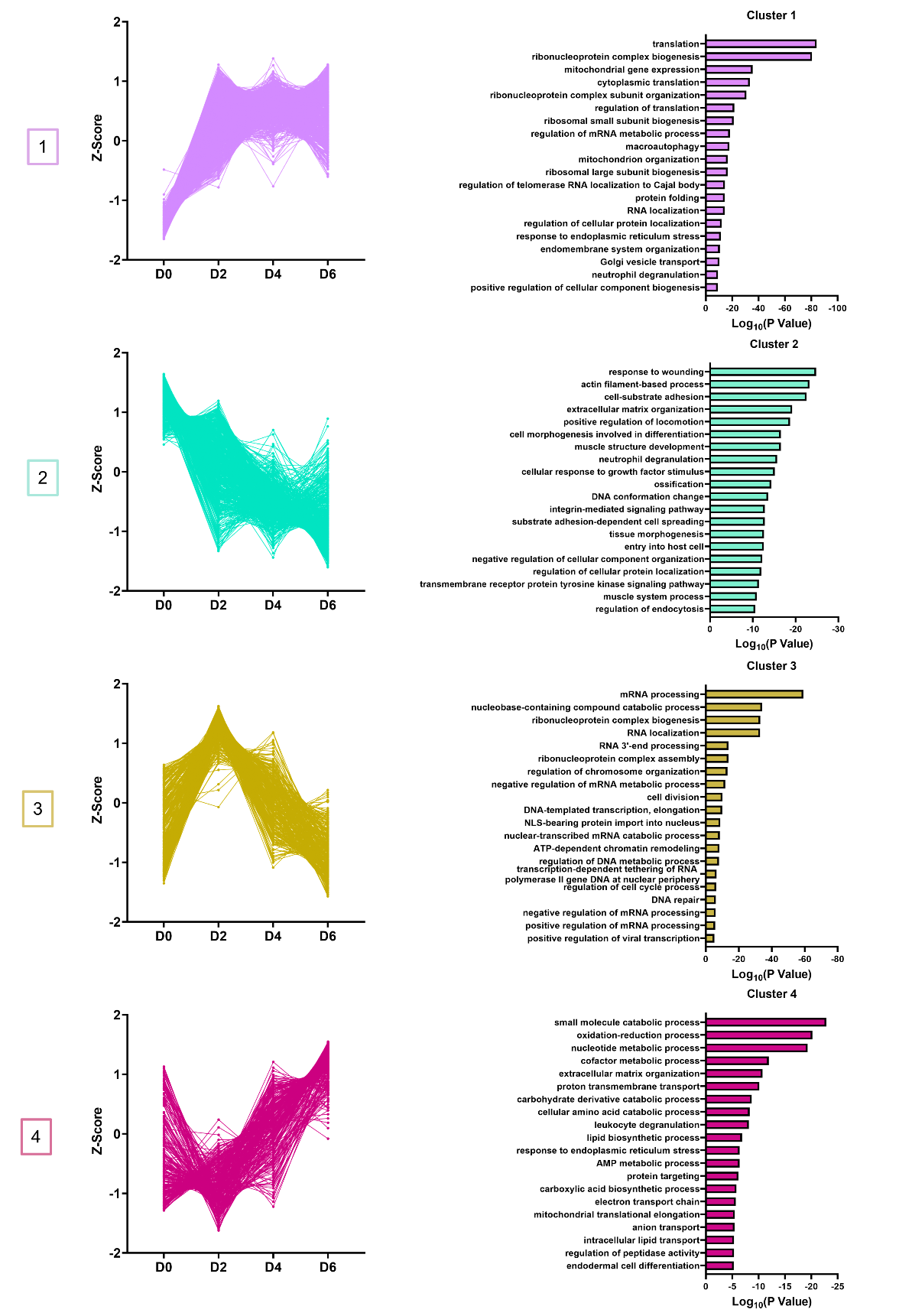
**

**Figure S2.**  Protein regulatory patterns and corresponding GO biological processes. Proteins from each cluster were plotted based on their Z-scored Log2(LFQ) at each time point. The top twenty enriched biological processes for each cluster with the highest –Log(P) values were plotted.

**
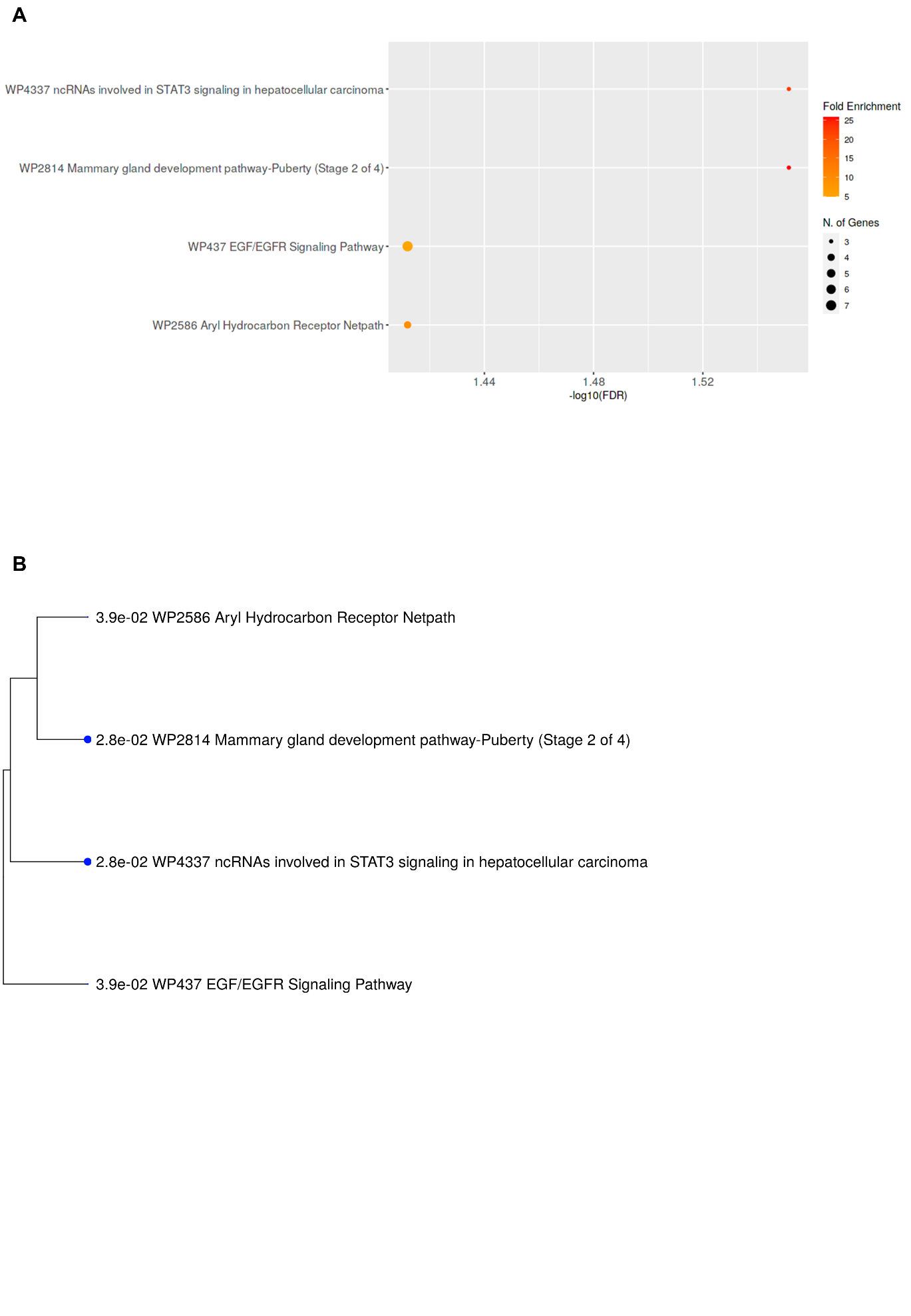
**

**Figure S3.** GO-BP of Newly identified phosphosites (**A, B**) GO Biological processes of proteins form which novel sites were identified in our dataset.

**
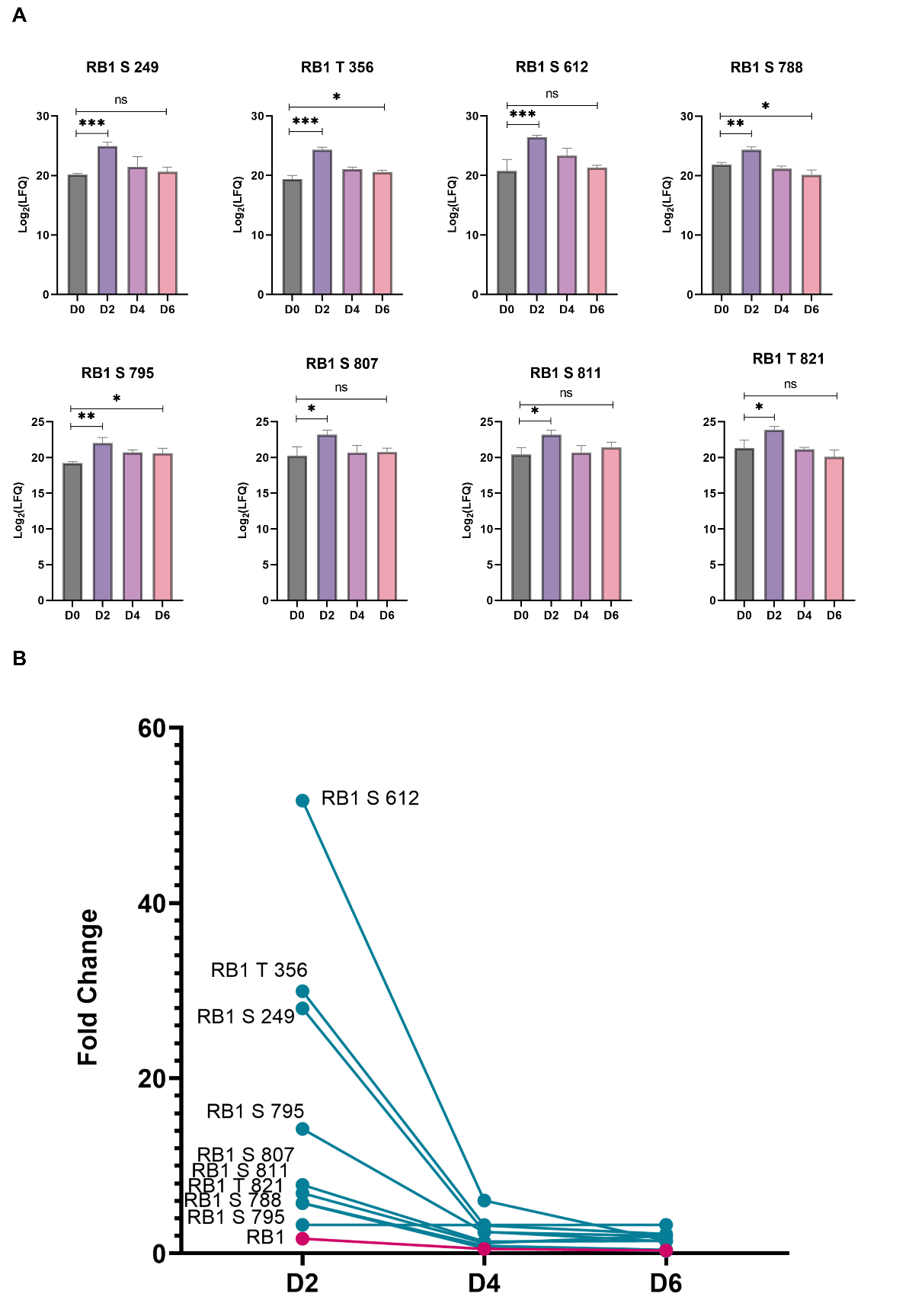
**

**Figure S4.** Dynamic regulation of Rb1 during cellular senescence. (**A**)Protein Rb1 phosphorylation during the progression of OIS. (**D**) Phosphorylation levels at each timepoint are compared to D0. Blue hue represents phosphosites, and pink hue represents overall protein levels.

**
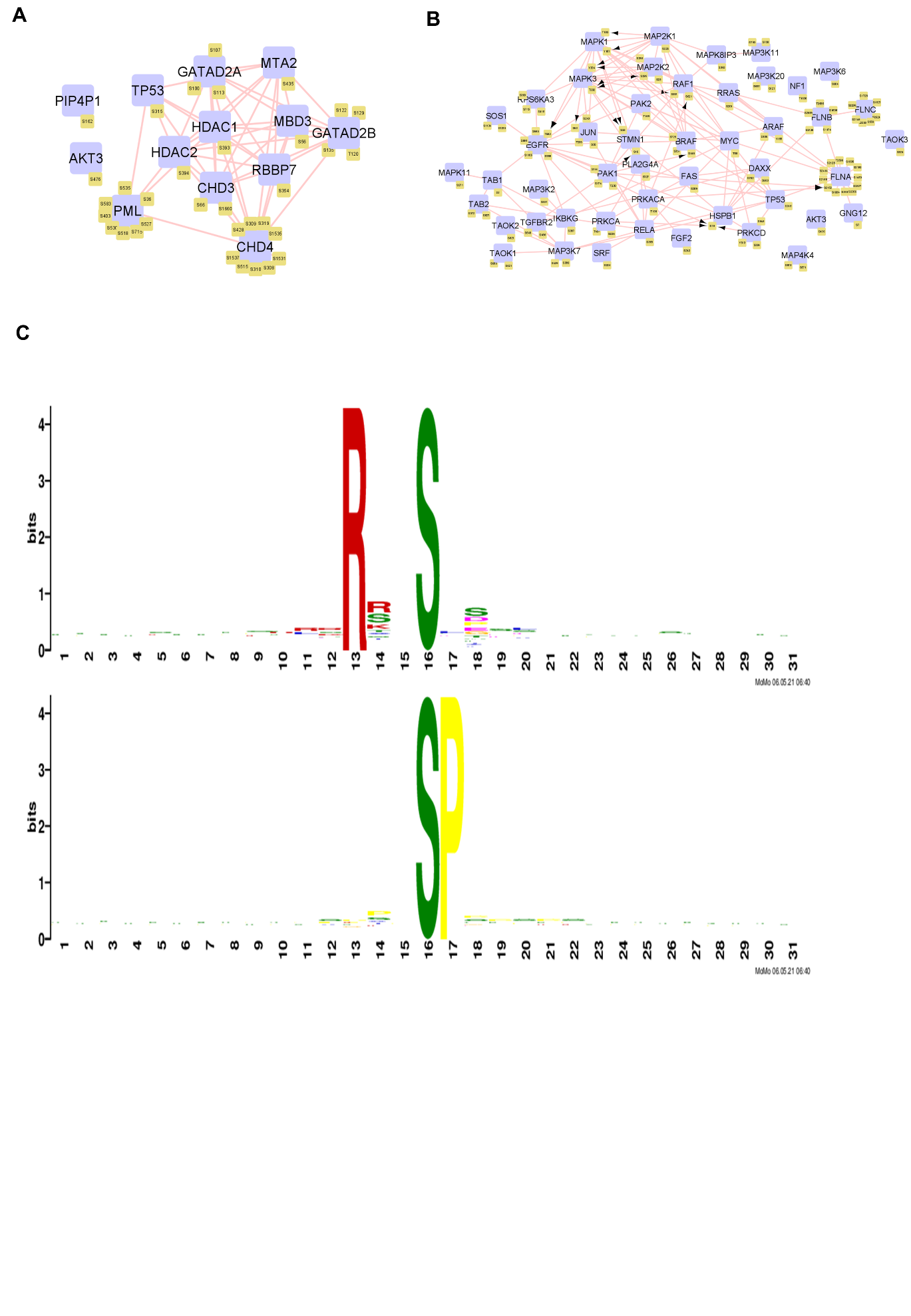
**

**Figure S5.** Nuclear phosphoproteomics of IMR90-ER:Ras. (**A**) Significantly regulated phosphosites involved in Regulation of TP53 Activity through Acetylation and (**B**) PTEN regulation. (**C**) Phosphorylated sequence motifs enriched at least 500 times.

**
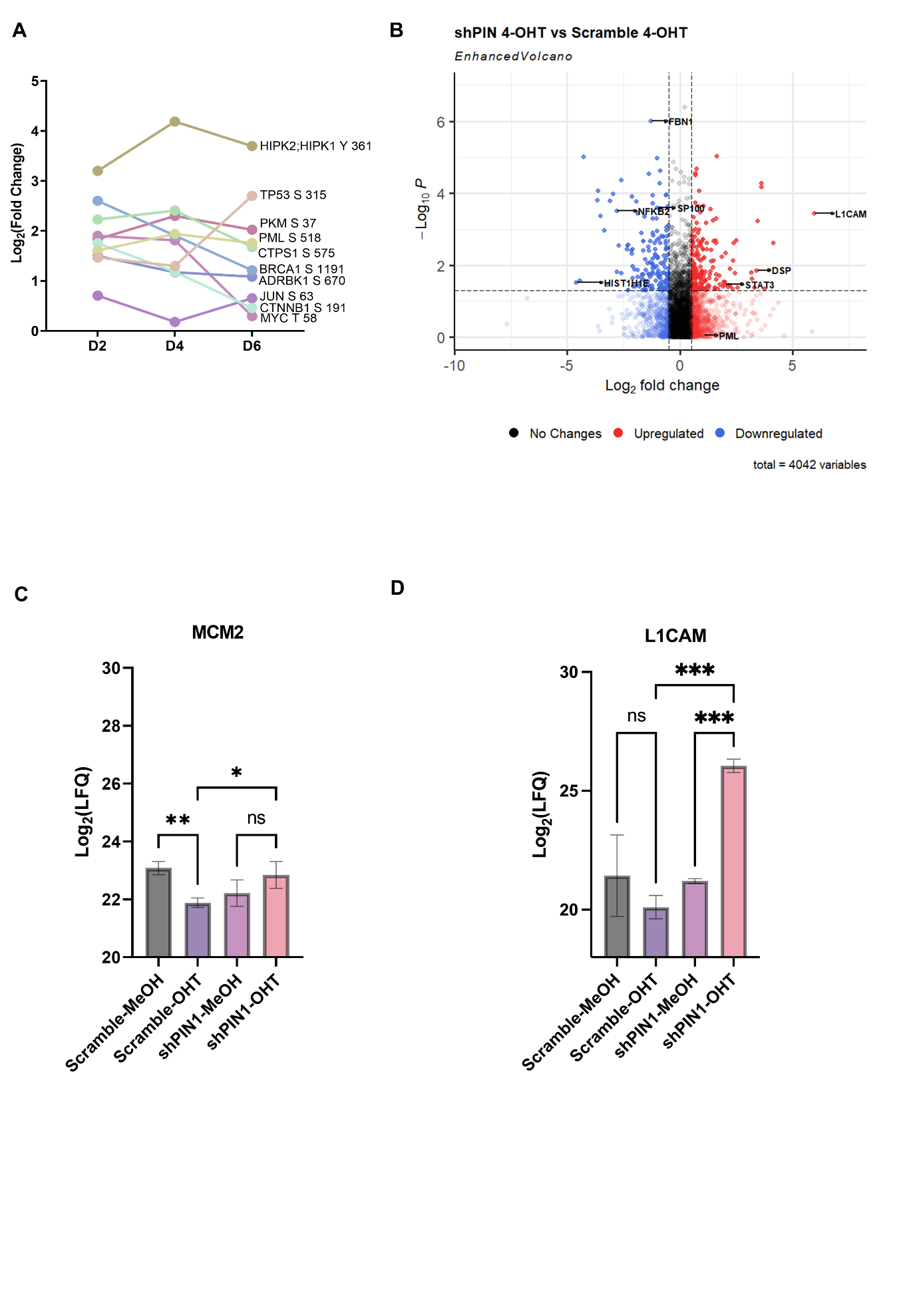

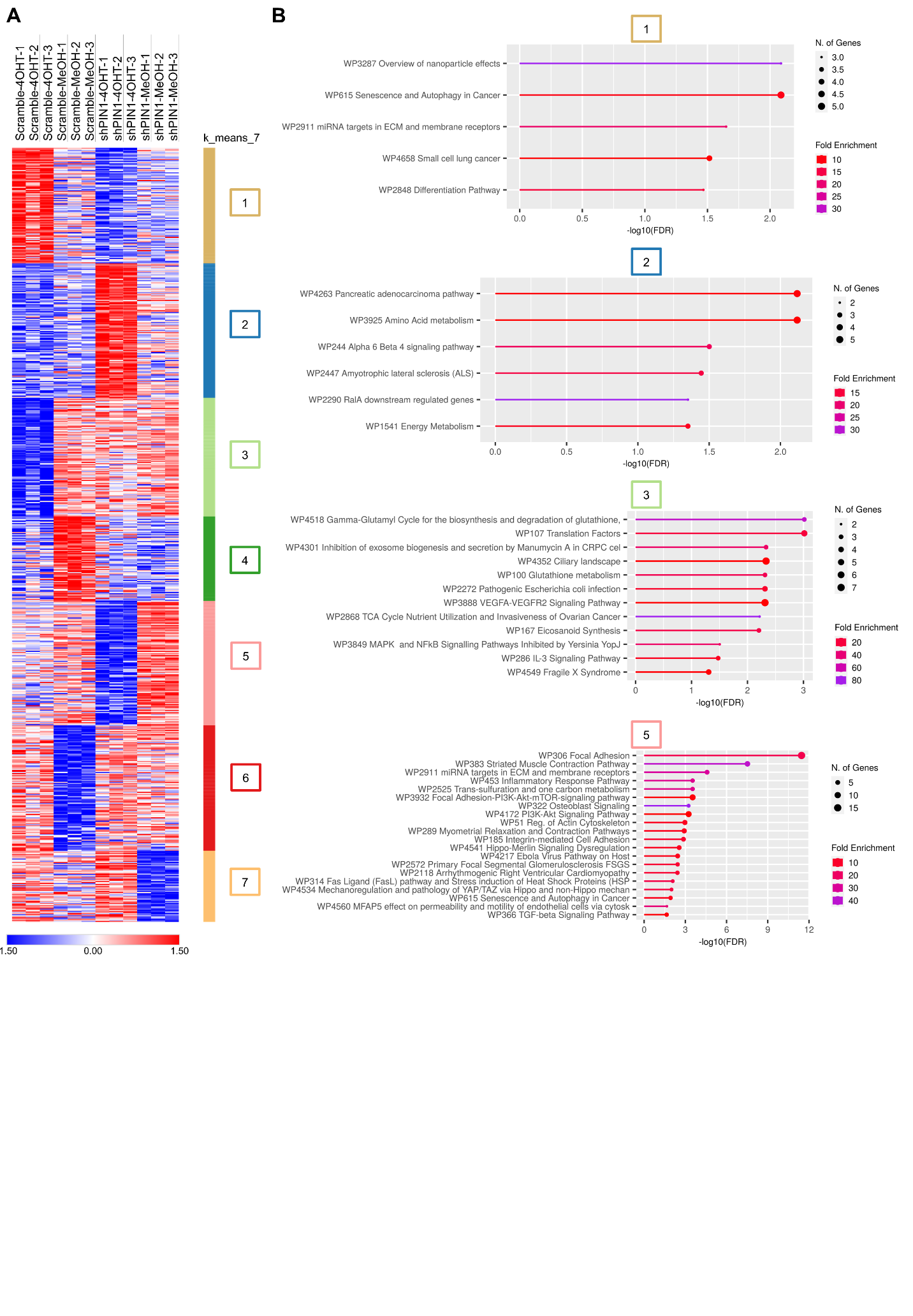
**

**Figure S6.** Pin1 regulated proteins. (**A**) Log2(Fold change) of PML-NB phosphosites that show PIN1 target motif. (**B**) Volcano plot representations of protein levels in response to Pin1 knockdown at 6 days of 4-OHT treatment. (**C**) Fold-change of proteins MCM2, (**D**) L1CAM.

**Figure S7**. shPIN1 proteome. (**A**) Heat map depicting the Z-scored Log2(LFQ) values of all significant proteins at each timepoint. Proteins were clustered by K-means clustering. Each cluster is indicated by a unique number and given distinct colors. Red hue indicates upregulated proteins, and blue hue indicates downregulated proteins. (**B**) Enriched GO-BP processes for clusters 1, 2, 3 and 5.

**
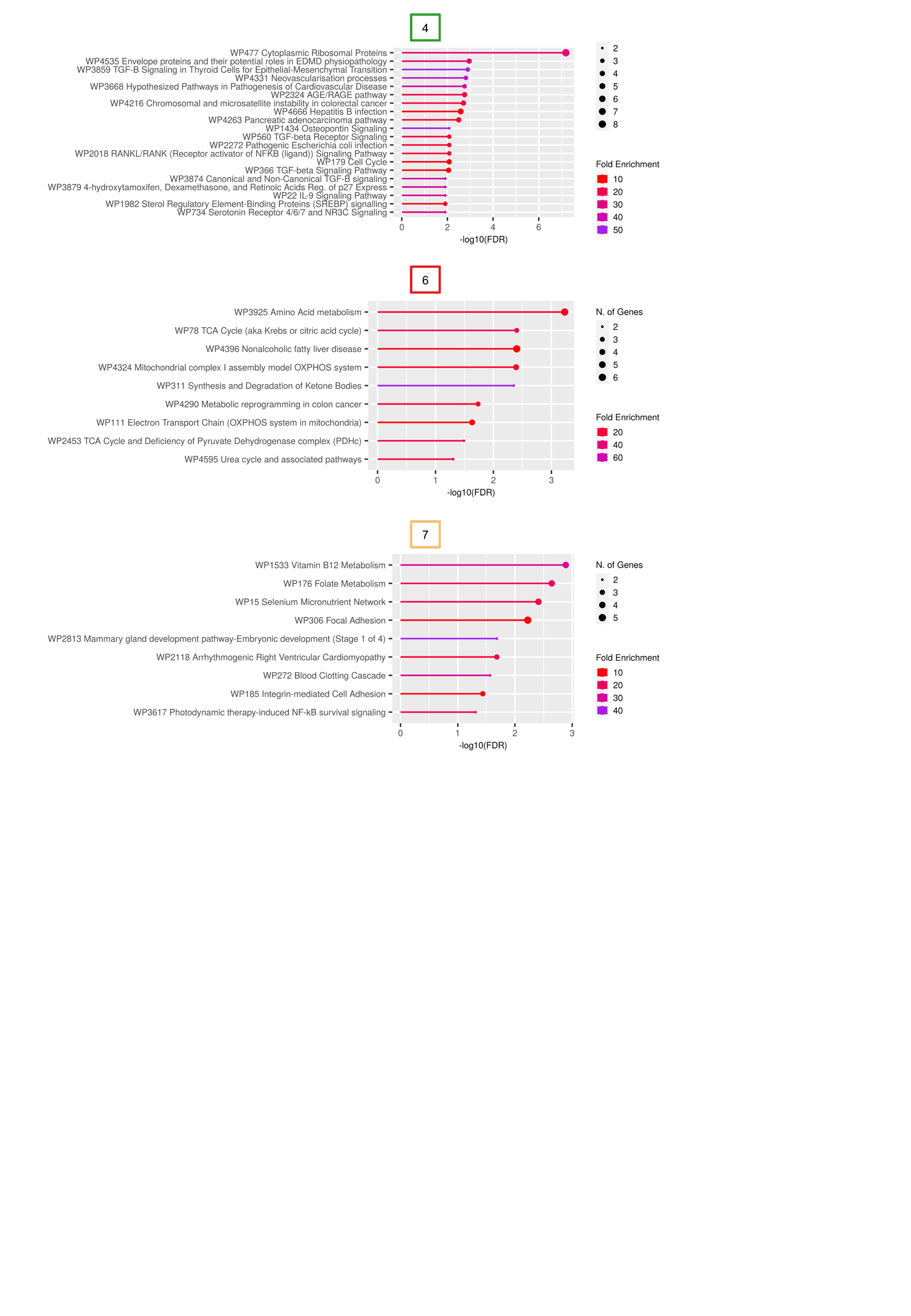
**

**Figure S8.** shPIN1 proteome, continued. (**A**) Enriched GO-BP processes for clusters 4, 6 and 7.
